# Rescuing Verubecestat: An Integrative Molecular Modeling and Simulation Approach for Designing Next-Generation BACE1 Inhibitors

**DOI:** 10.3390/ijms262412143

**Published:** 2025-12-17

**Authors:** Doni Dermawan, Nasser Alotaiq

**Affiliations:** 1 Department of Applied Biotechnology, Faculty of Chemistry, Warsaw University of Technology, 00-661 Warsaw, Poland; doni.dermawan.stud@pw.edu.pl; 2 Health Sciences Research Center (HSRC), Imam Mohammad Ibn Saud Islamic University (IMSIU), Riyadh 13317, Saudi Arabia

**Keywords:** Alzheimer’s disease, BACE1, frontier molecular orbital, molecular dynamics, pharmacophore, verubecestat

## Abstract

β-site amyloid precursor protein cleaving enzyme 1 (BACE1) is a central therapeutic target in Alzheimer’s disease, as it catalyzes the rate-limiting step in amyloid-β production. Verubecestat (VER), a clinical BACE1 inhibitor, failed in late-stage trials due to limited efficacy and safety concerns. This study employed an integrative computational approach to design VER derivatives with improved binding affinity, stability, and pharmacokinetic profiles. Structural similarity analysis, Molecular docking, frontier molecular orbital (FMO) analysis, pharmacophore modeling, 200 ns molecular dynamics (MD) simulations, MM/PBSA free energy calculations, and per-residue decomposition were performed. In silico ADMET profiling assessed drug-likeness, absorption, and safety parameters. Docking and pharmacophore analyses identified derivatives with stronger complementarity in the BACE1 catalytic pocket. MD simulations revealed that VERMOD-33 and VERMOD-57 maintained low root mean square deviations (RMSDs) and stable binding orientations and induced characteristic flexibility in the flap and catalytic loops surrounding the catalytic dyad (Asp93 and Asp289), consistent with inhibitory activity. MM/PBSA confirmed the superior binding free energies of VERMOD-33 (−51.12 kcal/mol) and VERMOD-57 (−43.85 kcal/mol), both outperforming native VER (−35.33 kcal/mol). Per-residue decomposition highlighted Asp93, Asp289, and adjacent flap residues as major energetic contributors. ADMET predictions indicated improved oral absorption, BBB penetration, and no mutagenicity or toxicity alerts. Rationally designed VER derivatives, particularly VERMOD-33 and VERMOD-57, displayed enhanced binding energetics, stable inhibitory dynamics, and favorable pharmacokinetic properties compared with native VER. These findings provide a computational framework for rescuing VER and support further synthesis and experimental validation of next-generation BACE1 inhibitors for Alzheimer’s disease.

## 1. Introduction

Alzheimer’s disease (AD) remains the most prevalent and devastating neurodegenerative disorder worldwide, affecting over 50 million people and projected to nearly triple by 2050 [1,2]. Clinically, AD is characterized by progressive cognitive decline, memory loss, and impaired daily functioning, with no available curative therapies [3]. Pathologically, the accumulation of amyloid-β (Aβ) peptides and tau neurofibrillary tangles is a hallmark of disease progression [4,5]. Among these, the dysregulated production of Aβ through sequential cleavage of amyloid precursor protein (APP) by β-site APP-cleaving enzyme 1 (BACE1) and γ-secretase has been recognized as a critical initiating event in AD pathogenesis [6,7]. Accordingly, pharmacological inhibition of BACE1 has emerged as an attractive therapeutic strategy aimed at reducing Aβ generation and slowing disease progression [8].

Over the past two decades, significant efforts have been devoted to the development of BACE1 inhibitors. Several small molecules demonstrated potent enzyme inhibition and robust Aβ reduction in preclinical studies, advancing into late-stage clinical trials [9,10]. However, enthusiasm was tempered when multiple candidates, including Verubecestat (MK-8931), Atabecestat, and Lanabecestat, failed in phase II/III trials due to safety concerns, poor tolerability, and lack of cognitive benefit [11,12]. Verubecestat (VER), in particular, was one of the most advanced BACE1 inhibitors, showing strong preclinical promise and favorable pharmacokinetics, including central nervous system (CNS) penetration [13,14]. Yet, despite its biochemical potency, clinical trials revealed dose-dependent adverse events such as neuropsychiatric symptoms, liver toxicity, and paradoxical worsening of cognitive function [15,16]. These outcomes highlight a central paradox: although BACE1 inhibition remains a validated therapeutic approach, existing inhibitors, including VER, are limited by off-target liabilities, suboptimal selectivity, and poor therapeutic indices.

Rational structural modification of VER represents a promising strategy to overcome these limitations. Specifically, fine-tuning its core scaffold to optimize BACE1 selectivity, improving blood–brain barrier (BBB) pharmacokinetics, and reducing off-target liabilities may yield next-generation inhibitors with enhanced clinical potential. Recent advances in computational drug design, including molecular docking, pharmacophore modeling, frontier molecular orbital (HOMO–LUMO) analysis, molecular dynamics (MD) simulations, free energy estimation, and in silico absorption, distribution, metabolism, excretion, and toxicity (ADMET) profiling, provide robust and cost-effective methodologies to systematically redesign and evaluate derivatives before experimental validation [17,18].

The present study aims to employ an integrative molecular modeling and simulation pipeline to rescue VER by designing and prioritizing novel derivatives with improved pharmacological and safety profiles. Using a combination of docking-based binding affinity screening against BACE1, HOMO–LUMO gap analysis, long-timescale MD simulations, MM/PBSA free energy estimation, and in silico ADMET prediction, we seek to identify modifications that preserve enzymatic potency while mitigating off-target effects. By integrating structural biology insights with modern cheminformatics, this work addresses the translational gap that hindered VER’s clinical success and provides a framework for developing next-generation BACE1 inhibitors. We hypothesize that rationally modified VER derivatives can achieve improved selectivity, optimized BBB penetration, and reduced systemic toxicity, thereby restoring the therapeutic promise of BACE1 inhibition in AD.

## 2. Results

### 2.1. Structure Alignment and Similarity Analysis

**Figure 1:**
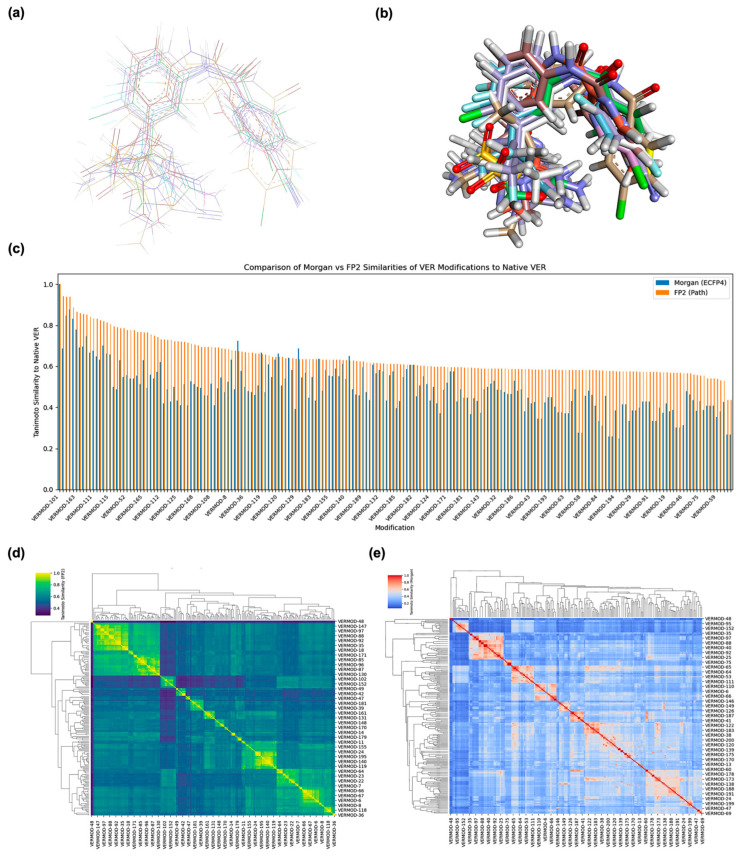
Structural alignment and similarity analysis of Verubecestat (VER) and its 200 designed derivatives. (**a**) 2D alignment of VER with representative derivatives, illustrating preservation of the central bicyclic core while allowing structural variation at peripheral substituents. (**b**) 3D conformational superposition of VER and derivatives, showing consistent alignment of the pharmacophoric core with divergent substituent orientations. (**c**) Comparison of Tanimoto similarity scores of each derivative to native VER using two molecular fingerprinting methods: FP2 (path-based, orange) and Morgan (ECFP4, blue), highlighting differences in sensitivity to local versus global structural changes. (**d**) Hierarchical clustering heatmap (FP2-based) of pairwise derivative similarities, where yellow regions indicate high similarity and dark blue regions indicate lower similarity. (**e**) Hierarchical clustering heatmap (Morgan ECFP4-based) of pairwise derivative similarities, where red regions denote high similarity clusters and blue regions reflect lower similarity relationships.

To ensure that the structural integrity of VER was preserved across its 200 designed derivatives, we performed a two-tiered assessment consisting of 3D structural alignment and fingerprint-based similarity analysis. This combined approach provided both a spatial and quantitative framework for evaluating scaffold conservation and modification diversity. As illustrated in Figure 1a, 2D structural alignment clearly showed that all 200 derivatives conserved the tricyclic pharmacophoric core of VER, a crucial determinant of its BACE1 inhibitory activity. Modifications were predominantly restricted to the peripheral substituents, including replacements on the aromatic rings and variations in the heteroaryl moieties. These changes were introduced to fine-tune physicochemical properties such as lipophilicity, polarity, and hydrogen-bonding capacity, while maintaining the essential binding geometry of VER. In 3D superposition analyses (Figure 1b), the conserved central scaffold aligned consistently across the derivative library, indicating that the designed analogs were chemically compatible with the parent backbone. The divergence was localized to the peripheral regions where substitutions extended into solvent-exposed or pocket-adjacent regions of the receptor-binding cleft. This spatial conservation was critical, as it suggests that the derivatives are likely to maintain productive anchoring within the BACE1 catalytic domain while exploring additional interaction hot spots.

To complement visual inspection, structural similarity was quantified using Tanimoto coefficients derived from two distinct fingerprinting strategies: FP2 (path-based fingerprints) and Morgan ECFP4 (circular fingerprints). The FP2 method primarily captures linear substructural paths, providing an overall scaffold-conservation metric, while Morgan fingerprints encode atom-centered circular environments, offering heightened sensitivity to localized structural modifications. In this study, FP2 and Morgan fingerprints were employed in a complementary manner to capture different dimensions of molecular similarity. The FP2 fingerprint is a path-based descriptor that encodes atom–bond sequences up to a defined length (typically seven bonds), making it well-suited for assessing global scaffold similarity and extended substructural continuity. In contrast, the Morgan (ECFP4) fingerprint is a circular atom–environment–based representation that encodes atomic neighborhoods within a fixed radius (two bonds), thereby emphasizing local structural variations such as heteroatom substitutions or small ring alterations [19]. These two methods provide complementary insights. FP2 reflects scaffold-level conservation, while Morgan captures localized chemical diversity, allowing for a balanced assessment of both global and fine-grained molecular relationships.

As shown in Figure 1c, the FP2 similarity distribution revealed a broad spectrum of conservation, with scores ranging from 0.43 to 0.94. This indicated that while certain derivatives (e.g., VERMOD-21, FP2 = 0.939) were nearly identical to the parent scaffold with only minor peripheral changes, others (e.g., VERMOD-48, FP2 = 0.447) represented more radical departures involving heteroaryl replacements or ring expansions. The Morgan fingerprint analysis reported a somewhat compressed distribution, generally yielding lower similarity scores, which reflects its heightened sensitivity to even subtle local modifications such as heteroatom substitutions or ring heterogeneity. This divergence between FP2 and Morgan scoring underscores the complementary perspectives offered by the two methods: FP2 excels in highlighting global scaffold conservation, whereas Morgan fingerprints better capture localized chemical innovations.

Hierarchical clustering of the similarity matrices offered a higher-order perspective on how the derivatives were grouped by modification category. In the FP2-based heatmap (Figure 1d), tightly clustered yellow regions corresponded to families of derivatives that shared common modification motifs, such as halogen substitutions on the phenyl ring or simple alkyl replacements. These clusters represented conservative modifications where scaffold preservation dominated. In contrast, derivatives that incorporated more disruptive features, such as bioisosteric replacements with heterocycles, electron-withdrawing group insertions, or dual-ring fusions, segregated into darker blue regions, indicating significant deviation from the parent scaffold. The Morgan ECFP4 clustering (Figure 1e) provided a more granular classification. While major modification categories were still apparent, the matrix showed a more fragmented similarity landscape, with subclusters reflecting specific substitution patterns such as fluorinated heteroaryls, alkoxy-bearing aromatics, and extended π-conjugated systems. This suggests that Morgan fingerprints were more effective at capturing the nuanced chemical features introduced in VER derivatives.

Selected representatives summarized in Table 1 further highlight the diversity of modifications. For instance, VERMOD-72 (halogen substitution, FP2 similarity = 0.78) retained moderate similarity, suggesting preserved binding potential while exploring halogen-mediated interactions with hydrophobic pockets. In defining acceptable similarity thresholds, we followed established cheminformatics conventions in which Tanimoto coefficients (Tc) ≥ 0.85 denote high structural similarity, implying close analog relationships, while 0.50 ≤ Tc < 0.85 represents moderate similarity, indicating retention of key pharmacophoric elements with permissible modifications. Given that most designed derivatives fall within the 0.56–0.78 range, our results indicate a moderate-to-high level of scaffold preservation consistent with the intended goal of maintaining the Verubecestat core while introducing chemical diversity at peripheral sites. This similarity window provides an optimal balance between molecular novelty and pharmacophoric continuity, supporting meaningful structure–activity relationship (SAR) exploration without redundancy. VERMOD-33 (alkyl substitution, FP2 = 0.56) exhibited a lower similarity score, reflecting bulkier substituents that may impact steric complementarity with BACE1. VERMOD-94 (heterocyclic substitution, FP2 = 0.57) represented a bioisosteric approach, likely altering hydrogen-bonding patterns and electronic distribution within the active site. At the other extreme, VERMOD-198 (FP2 = 0.89) and VERMOD-21 (FP2 = 0.94) retained near-native similarity, suggesting high potential to maintain VER’s binding orientation while subtly optimizing physicochemical traits. These findings suggest a balanced design strategy: the conserved clusters (high similarity scores) are valuable for maintaining anchoring interactions within the catalytic aspartyl dyad of BACE1, while the divergent clusters introduce novel substituents that may exploit secondary binding pockets, improve pharmacokinetics, or reduce off-target toxicity. By mapping the structural conservation and divergence landscape across 200 analogs, this alignment and similarity analysis establishes a robust foundation for downstream docking simulations, binding free energy evaluations, and ADMET predictions. Ultimately, the dual emphasis on scaffold preservation and structural innovation ensures that VER modifications are both mechanistically grounded and chemically diverse, providing fertile ground for rescuing the clinical potential of this once-promising BACE1 inhibitor.

### 2.2. Molecular Docking Results, Binding Pose, and Binding Affinity Analysis

**Figure 2:**
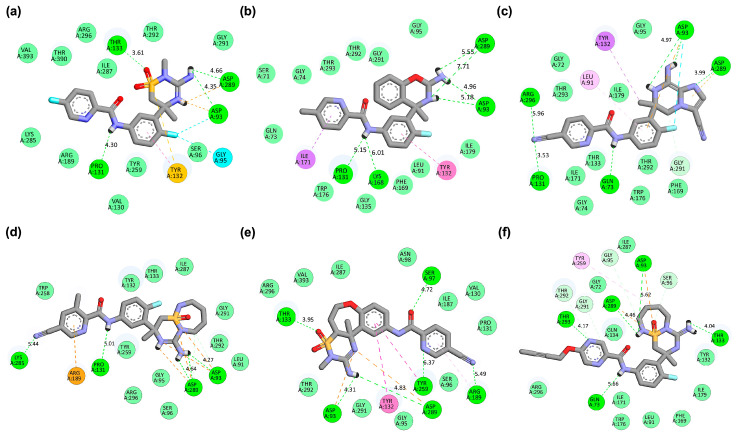
Two-dimensional interaction maps of Verubecestat (VER) and its best-performing derivatives with the BACE1 binding site. (**a**) BACE1_VER complex. (**b**) BACE1_VERMOD-33 complex. (**c**) BACE1_VERMOD-57 complex. (**d**) BACE1_VERMOD-9 complex. (**e**) BACE1_VERMOD-10 complex. (**f**) BACE1_VERMOD-168 complex. The interaction types are color-coded as follows: hydrogen bonds (bright green), carbon-hydrogen bonds (light green), van der Waals interactions (pale green), Pi-Alkyl (pink), and Pi-Sigma (purple).

Molecular docking was employed to evaluate the binding potential of VER and a series of its designed derivatives against the BACE1 catalytic domain. The docking protocol provided both quantitative estimates of binding affinity and qualitative insights into the interaction patterns within the active site. As expected, native VER demonstrated a reproducible binding conformation with a HADDOCK score of −44.1 ± 0.5 a.u. and a free binding energy of −8.44 kcal/mol. Its pose was stabilized by hydrogen bonds with Asp93, Pro131, Thr133, and Asp289, residues that form the canonical interaction framework for potent BACE1 inhibition (Table 2). Among these, Asp93 and Asp289 represent the aspartyl dyad crucial for catalysis, while Thr133 and Pro131 contribute to maintaining structural complementarity and orientation of the inhibitor within the flap-binding pocket (Figure 2a). This characteristic interaction fingerprint of VER served as the benchmark for evaluating derivative performance.

A notable trend emerged among the derivatives: those that achieved enhanced docking scores and lower binding free energies while conserving VER’s canonical hydrogen bonds tended to outperform energetically competitive analogs that deviated from the native interaction profile. For instance, VERMOD-33 emerged as a strong candidate, with the most favorable free binding energy (−10.42 kcal/mol) among the derivatives. Its docking map (Figure 2b) revealed hydrogen bonds with Asp93, Pro131, and Asp289, while also engaging Lys168, a residue located in the flap region adjacent to the active site. This interaction is particularly relevant, as flap stabilization has been associated with increased inhibitor residence time and improved therapeutic efficacy. This interaction is particularly relevant because stabilization of the flap region has been correlated with increased inhibitor residence time, which reflects how long an inhibitor remains bound before dissociating from the target enzyme. Although direct residence time cannot be experimentally measured from docking simulations, it can be inferred computationally from molecular dynamics (MD) simulations by monitoring the stability and lifetime of key protein–ligand contacts, such as hydrogen bonds and hydrophobic interactions, over the course of the trajectory. Specifically, sustained binding to flap-associated residues (e.g., Lys168 and Thr133) during MD sampling serves as a kinetic proxy, indicating slower dissociation tendencies and extended residence behavior. The balance between canonical hydrogen bonds and flap-targeted interactions positions VERMOD-33 as a promising analog that could exhibit both strong affinity and favorable binding kinetics in dynamic simulations.

Similarly, VERMOD-57 recorded the most favorable HADDOCK score (−62.2 ± 0.7 a.u.) and a significantly improved binding energy (−10.11 kcal/mol). Its binding orientation (Figure 2c) overlapped extensively with VER’s pharmacophore but further reinforced stability by introducing additional hydrogen bonds with Gln73 and Arg296. Importantly, these new interactions did not compromise the hydrogen bonds to Asp93, Pro131, and Asp289, thereby extending the interaction network while preserving the inhibitory core. This extended network likely contributes to longer contact persistence times during MD simulations, which are indicative of improved kinetic stability and longer inhibitor residence within the BACE1 active pocket. This hybrid profile suggests that VERMOD-57 benefits from a combination of conserved pharmacophoric anchoring and augmented electrostatic stabilization, a hallmark of rationally optimized inhibitors.

VERMOD-9 also demonstrated improved affinity relative to VER, with a HADDOCK score of −54.9 ± 0.5 a.u. and a binding energy of −9.79 kcal/mol. Its hydrogen bonding profile (Figure 2d) included interactions with Asp93, Pro131, and Asp289, consistent with the canonical pattern, while introducing an additional hydrogen bond with Lys285. This modification illustrates how subtle structural changes can extend ligand reach to peripheral residues without disrupting the fundamental inhibitory framework. The inclusion of Lys285 likely contributes to the reduced desolvation penalty observed for VERMOD-9 compared to VER, offering a thermodynamic advantage. In contrast, VERMOD-10 and VERMOD-168, while still exhibiting competitive docking scores (−52.7 and −51.8 a.u., respectively) and binding energies (both around −9.0 kcal/mol), deviated from the canonical VER binding fingerprint. VERMOD-10 (Figure 2e) formed hydrogen bonds with Ser97, Arg189, and Tyr259, while retaining partial overlap at Asp93 and Asp289. Similarly, VERMOD-168 (Figure 2f) displayed interactions with Gln73 and Thr293, but its reliance on Thr133 and Asp289 was diminished compared to VER. Although these derivatives achieved strong energetic profiles, the shift in hydrogen bonding networks raises concerns about their inhibitory specificity and long-term stability within the catalytic pocket. Such deviations underscore that affinity alone does not guarantee pharmacophoric fidelity, and maintaining interactions with the catalytic dyad remains essential for preserving mechanistic relevance.

**Figure 3:**
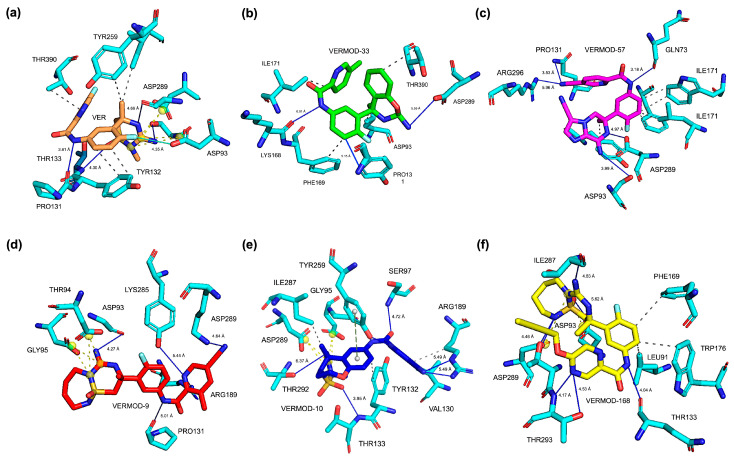
Comparative 3D binding poses of Verubecestat (VER) and its best-performing derivatives in the BACE1 ligand-binding domain (LBD). (**a**) BACE1_VER complex. (**b**) BACE1_VERMOD-33 complex. (**c**) BACE1_VERMOD-57 complex. (**d**) BACE1_VERMOD-9 complex. (**e**) BACE1_VERMOD-10 complex. (**f**) BACE1_VERMOD-168 complex.

The 3D binding pose analysis revealed that native VER occupies the BACE1 active site in a compact and deeply buried orientation (Figure 3a), with its central scaffold aligned toward the catalytic aspartyl dyad (Asp93 and Asp289) while extending peripheral substituents toward the flap loop (Tyr132–Thr133 region). This spatial arrangement ensures simultaneous stabilization by hydrogen bonding to the dyad and steric accommodation within the S1 pocket, explaining its reliable inhibitory activity. In contrast, derivatives such as VERMOD-33 and VERMOD-57 adopted slightly rotated orientations, which extended their substituents toward auxiliary binding regions, thereby enhancing the breadth of their interaction networks. Specifically, VERMOD-33 projected one aromatic moiety toward the Lys168/Phe169 zone of the flap, while still anchoring to Asp93 and Asp289, thereby broadening the binding interface (Figure 3b). Meanwhile, VERMOD-57 penetrated deeper into the active site cleft (Figure 3c), with one polar substituent reorienting toward Gln73 and Arg296, residues positioned beyond the catalytic core, generating an expanded hydrogen-bonding network that compensated for conformational rearrangements in the dyad region.

By contrast, VERMOD-9, VERMOD-10, and VERMOD-168 displayed more variable orientations, highlighting the role of ligand flexibility in defining binding stability. VERMOD-9 assumed an elongated pose along the binding groove (Figure 3d), maintaining alignment with the catalytic dyad but extending outward toward Lys285 and Arg189, suggesting improved interactions with solvent-exposed residues. VERMOD-10 exhibited a partially shifted positioning (Figure 3e) that tilted its heteroaromatic core closer to Thr94 and Gly95, while its peripheral substituents reached toward Arg189, implying potential stabilization via flap-loop interactions despite reduced overlap with the dyad. Lastly, VERMOD-168 rotated further within the LBD (Figure 3f), situating its bulky substituents near Leu91, Phe169, and Trp176, creating additional van der Waals contacts, but at the cost of weakening its canonical hydrogen bond network with the dyad. These variations demonstrate how subtle scaffold modifications redirect ligands across distinct sub-pockets of BACE1, influencing not only energetic profiles but also the pharmacophoric fidelity of dyad engagement.

The molecular interaction profiles highlight distinct patterns of atom–atom contacts between BACE1 and the studied ligands, reflecting how scaffold modifications influence binding stabilization (Table 3). Native VER (BACE1_VER complex) established a balanced interaction network, with moderate carbon–carbon (CC = 1770) and carbon–nitrogen (CN = 1028) contacts, complemented by a considerable number of carbon–oxygen (CO = 818) and nitrogen–oxygen (NO = 254) interactions. This profile underscores VER’s compact yet efficient binding, with interactions centered primarily around the catalytic dyad and flap loop. Importantly, the relatively high CC and CN counts suggest stable hydrophobic packing and polar interactions, which underpin its known inhibitory activity. However, the lower overall interaction totals compared to derivatives indicate that VER’s native scaffold may not fully exploit the breadth of sub-pockets available in the BACE1 binding domain.

Among the derivatives, VERMOD-33 and VERMOD-57 exhibited substantial increases in total interactions, with VERMOD-33 showing the highest CC count (2454) and balanced contributions from CO (1000) and CN (1083). This reflects an expanded hydrophobic footprint while maintaining sufficient polar contacts, consistent with its rotated pose toward Lys168/Phe169 observed in the structural analysis. Similarly, VERMOD-57 demonstrated a striking rise in CN (1484) and NO (330) contacts, accompanied by significant NN (230) contributions, suggesting that its deeper penetration into the active cleft promoted extensive polar and hydrogen-bond-like interactions. These results reinforce the structural interpretation that VERMOD-57 leverages auxiliary binding residues such as Gln73 and Arg296 to broaden its stabilization network. Other derivatives displayed more nuanced interaction profiles. VERMOD-9, while generating higher CN (1216) and NO (271) contacts than native VER, presented lower CC counts (1991) compared to VERMOD-33 and VERMOD-57, reflecting a more elongated pose that partially extended into solvent-accessible regions rather than maximizing hydrophobic enclosure. VERMOD-10, by contrast, generated exceptionally high CO interactions (1100), alongside strong CN (1216) and NO (320) counts, pointing to an orientation that favored oxygen-rich polar stabilization near Thr94 and Gly95. Finally, VERMOD-168 emerged as one of the most interaction-rich derivatives, with robust CC (2431), CN (1428), and NO (332) contributions, as well as notable CO (1149) values. This suggests that despite its rotated orientation, the bulky scaffold of VERMOD-168 successfully occupied multiple sub-pockets, reinforcing both hydrophobic packing and polar complementarity.

### 2.3. Frontier Molecular Orbital (HOMO–LUMO) Results of Verubecestat and Its Derivatives

**Figure 4:**
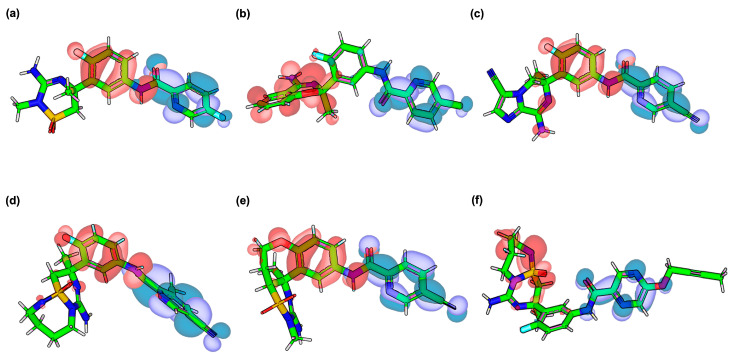
HOMO–LUMO profiles of Verubecestat (VER) and its best-performing derivatives. (**a**) VER. (**b**) VERMOD-33. (**c**) VERMOD-57. (**d**) VERMOD-9. (**e**) VERMOD-10. (**f**) VERMOD-168. Blue regions represent HOMO orbital localization, while red regions denote LUMO orbital distribution.

The frontier molecular orbital (FMO) analysis provides valuable insights into the electronic structure of VER and its top-performing derivatives, highlighting the regions of electron donation (HOMO) and acceptance (LUMO) that may govern molecular reactivity and binding to BACE1 (Table 4). As shown in Figure 4a, the HOMO of VER is mainly localized around the triazine heteroaromatic core and its adjacent nitrogen substituents, which are electron-rich and capable of engaging in hydrogen bonding or electrostatic interactions with the catalytic dyad residues of BACE1. In contrast, the LUMO is distributed over the fluorophenyl and oxetane moieties, suggesting that these distal substituents can participate in electron-accepting processes and stabilize π-stacking or dipole interactions within the hydrophobic pocket. Although the HOMO and LUMO orbitals display limited direct spatial overlap, this separation does not negate the possibility of charge transfer; rather, it indicates an intramolecular charge transfer (ICT) mechanism where electron density migrates from donor-rich (HOMO) regions to electron-deficient (LUMO) moieties upon excitation. Such donor–acceptor separation-driven transitions are well-documented in heteroatom-rich, asymmetric drug-like scaffolds and represent through-space rather than overlap-dependent charge transfer behavior. This spatial segregation, combined with an energy-level offset, supports a directional electron redistribution pathway that may facilitate efficient binding stabilization.

For the derivatives, distinct HOMO–LUMO redistribution patterns were observed, correlating with their enhanced binding affinities. In VERMOD-33 (Figure 4b), the HOMO extends further across the aromatic linker compared to VER, while the LUMO is concentrated near the terminal heteroaryl substituent, producing a reduced gap (3.87 eV) that indicates improved electronic flexibility. Here, the partial segregation of HOMO (on the triazine–amide region) and LUMO (on the chloropyridine moiety) suggests a localized charge migration channel that may enhance donor–acceptor communication across the conjugated framework. VERMOD-57 (Figure 4c) exhibits a more delocalized HOMO across the triazine and fluorophenyl units, while its LUMO is lowered in energy (−2.71 eV), reducing the HOMO–LUMO gap to 3.74 eV. Although HOMO–LUMO overlap remains moderate, their complementary localization (HOMO on the donor region, LUMO on the cyano-substituted acceptor) indicates a long-range charge transfer process. This enhanced polarizability may underlie the strong interaction network observed in docking and MD simulations.

In VERMOD-9 (Figure 4d), the HOMO density is shifted toward the oxetane-substituted region, while the LUMO resides on the extended aromatic substituent, resulting in one of the lowest HOMO–LUMO gaps (3.62 eV). The enhanced dipole moment (4.43 D) suggests improved orientation-dependent interactions with BACE1 residues. Similarly, VERMOD-10 (Figure 4e) demonstrates the smallest gap among all derivatives (3.29 eV), with the HOMO spread over the heteroaromatic scaffold and the LUMO directed toward the polar substituents. This orbital arrangement, combined with the highest dipole moment (5.13 D), supports its strong electronic adaptability within the enzyme’s binding pocket. Finally, VERMOD-168 (Figure 4f) presents a HOMO predominantly on the triazine ring and a LUMO localized on the substituted phenyl group, yielding a moderate gap (3.93 eV). Although its dipole moment (1.80 D) is lower than most derivatives, the orbital distribution reflects a balance between electron-donating and electron-accepting centers, which may support stable hydrophobic and hydrogen-bond interactions. Overall, compared to VER, the derivatives display narrower HOMO–LUMO gaps and, in several cases, higher dipole moments, both of which are indicative of increased electronic reactivity and adaptability to the BACE1 active site environment.

### 2.4. Pharmacophore Modeling Results

**Figure 5:**
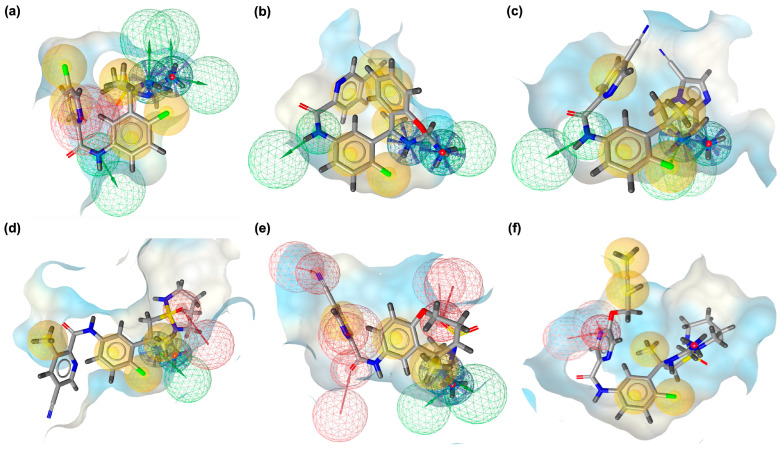
Three-dimensional pharmacophore modeling. (**a**) BACE1_VER complex. (**b**) BACE1_VERMOD-33 complex. (**c**) BACE1_VERMOD-57 complex. (**d**) BACE1_VERMOD-9 complex. (**e**) BACE1_VERMOD-10 complex. (**f**) BACE1_VERMOD-168 complex. Yellow spheres indicate hydrophobic interactions, green arrows represent hydrogen bond donors, and red arrows signify hydrogen bond acceptors.

Pharmacophore modeling was employed to rationalize the docking and FMO findings by identifying the critical interaction features driving recognition between BACE1 and VER or its derivatives (Figure 5). The pharmacophore models revealed distinct distributions of hydrogen bond donors, hydrogen bond acceptors, and hydrophobic sites, which together establish the unique interaction signatures of VER and its derivatives (Figure 5). These models help explain why certain scaffold modifications improved stabilization within the catalytic pocket while others reoriented interactions toward auxiliary sub-pockets. In the native BACE1_VER complex (Figure 5a), the pharmacophore model is relatively compact, dominated by balanced hydrophobic contacts (yellow spheres) surrounding the triazine core, and moderate donor/acceptor contributions positioned near the catalytic dyad. This reflects the efficient yet spatially limited recognition pattern of VER, consistent with its moderate CC and CN interaction counts. However, the limited spread of pharmacophoric features suggests that VER underutilizes available sub-pockets, restricting its interaction versatility compared to derivatives. For VERMOD-33 (Figure 5b), the pharmacophore model shows expanded hydrophobic regions along the aromatic scaffold, accompanied by strategically positioned acceptors. This expanded footprint supports the docking-derived interpretation that VERMOD-33 exploits deeper hydrophobic sub-pockets, especially near Lys168 and Phe169, while still retaining essential polar stabilization. The pharmacophore alignment thus explains its high CC count and balanced CO/CN contributions.

In the case of VERMOD-57 (Figure 5c), the model reveals a strong enrichment of hydrogen bond acceptors and donors, particularly oriented toward the deeper cleft of the catalytic site. The denser donor/acceptor network explains the high CN and NO counts observed in the interaction profile, as well as its reduced HOMO–LUMO gap and enhanced polarizability. This pharmacophore organization underscores why VERMOD-57 is able to recruit auxiliary residues such as Gln73 and Arg296 for additional stabilization. VERMOD-9 (Figure 5d) presents a more elongated pharmacophore distribution, with hydrogen bond acceptors positioned at the solvent-exposed tail and hydrophobic moieties retained near the triazine region. While this explains the enhanced CN/NO contacts, the weaker hydrophobic enclosure (lower CC count) matches the more solvent-accessible orientation derived from docking. The pharmacophore thus highlights its trade-off between polar complementarity and compact hydrophobic stabilization.

The pharmacophore of VERMOD-10 (Figure 5e) is rich in hydrogen bond acceptors, particularly clustered near its oxygen-containing substituents. This dense network of acceptors accounts for the exceptionally high CO and NO contacts observed in docking, as well as its strong dipole moment revealed by FMO analysis. The pharmacophore confirms that VERMOD-10 adopts an interaction strategy heavily reliant on oxygen-driven polar stabilization near Thr94 and Gly95, maximizing electronic adaptability within the active site. Finally, VERMOD-168 (Figure 5f) displays a broad pharmacophore coverage, with hydrophobic features dominating across the bulky scaffold and acceptors/donors distributed to support multiple binding orientations. This wide-ranging pharmacophore arrangement rationalizes the interaction-rich profile of VERMOD-168, with simultaneous reinforcement of CC, CN, and NO contacts. Although its dipole moment is lower than that of other derivatives, the spatial diversity of its pharmacophore features enables occupation of multiple sub-pockets, resulting in a robust stabilization network.

### 2.5. MD Simulations Reveal Structural Stability and Interaction Profiles of Verubecestat and Its Derivatives

**Figure 6:**
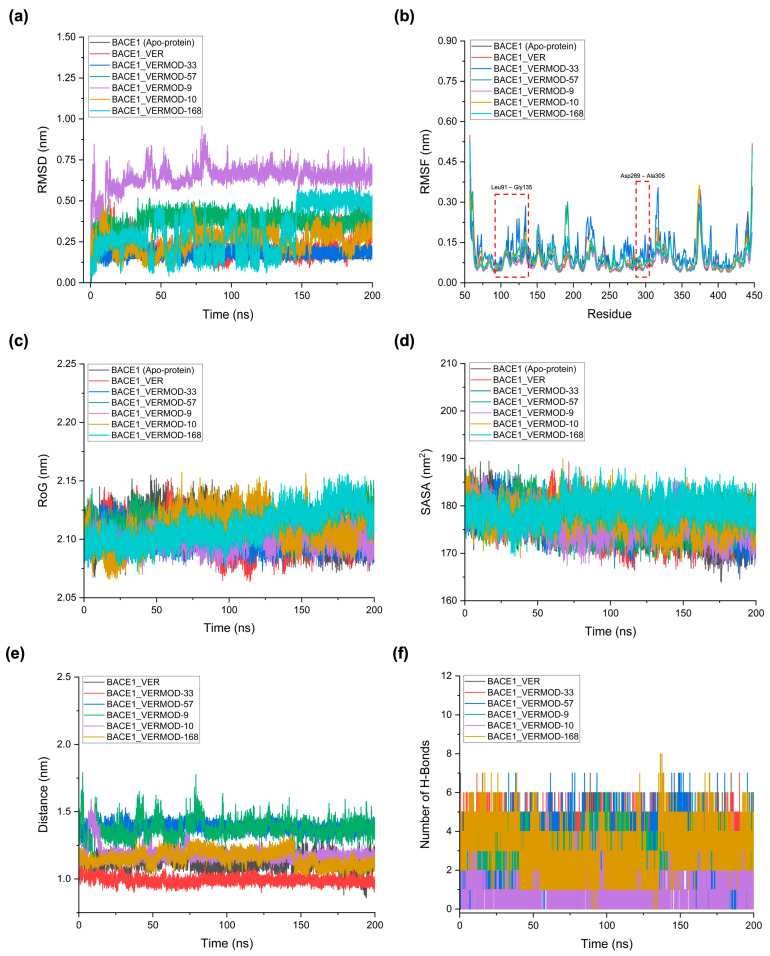
MD simulation results for BACE1 in complex with Verubecestat (VER) and top-performing derivatives over 200 ns of simulation. (**a**) Root mean square deviation (RMSD), reflecting the overall conformational stability of the protein–ligand complexes. (**b**) Root mean square fluctuation (RMSF) provides residue-level insights into backbone flexibility, particularly in the active-site regions. (**c**) Radius of gyration (RoG), indicating the degree of compactness and folding stability of the protein throughout the trajectory. (**d**) Solvent accessible surface area (SASA), showing changes in surface exposure and solvation upon ligand binding. (**e**) Ligand–protein center-of-mass distance, illustrating the persistence and dynamic retention of ligands within the binding cavity. (**f**) The number of hydrogen bonds represents the occupancy and stability of polar contacts sustaining protein–ligand interactions.

To assess the dynamic behavior and stability of BACE1 in complex with VER and the top-performing derivatives, 200 ns MD simulations were performed. The analysis included root mean square deviation (RMSD), root mean square fluctuation (RMSF), radius of gyration (RoG), solvent-accessible surface area (SASA), ligand–protein center-of-mass distance, and hydrogen bond profiles (Table 5, Figure 6). RMSD analysis (Figure 6a) highlighted distinct stability profiles across the studied complexes. The apo-BACE1 exhibited the lowest deviation (0.15 nm), reflecting intrinsic conformational stability in the absence of ligands. The binding of native VER increased the RMSD to 0.21 nm, indicative of modest structural adjustments upon ligand accommodation. Among the derivatives, VERMOD-33 demonstrated stable behavior with a mean RMSD of 0.18 nm, even lower than native VER, suggesting enhanced stabilization of the protein–ligand complex. By contrast, VERMOD-9 and VERMOD-57 showed higher RMSD values (0.64 nm and 0.37 nm, respectively), reflecting larger conformational rearrangements associated with their elongated or deeply penetrating binding orientations. VERMOD-168 and VERMOD-10 displayed intermediate stability, with average RMSDs of 0.31 nm and 0.26 nm, respectively. These results indicate that scaffold modifications can either stabilize (e.g., VERMOD-33) or destabilize (e.g., VERMOD-9) the complex, depending on their fit within the binding cleft.

RMSF profiles (Figure 6b) provided residue-level insights into the local flexibility of BACE1 in different simulation systems. Compared to the apo protein, all ligand-bound complexes demonstrated increased fluctuations in the loop regions spanning Leu91–Gly135 and Asp289–Ala305, which flank the active site pocket. These regions correspond to the flexible flap loop (residues ~ 90–135), which controls access to the catalytic cavity, and the catalytic loop containing the Asp289–Asp305 dyad, which plays a central role in substrate recognition and cleavage. In the apo form, these loops remained relatively stable, stabilized by a network of intramolecular hydrogen bonds. Upon ligand binding, however, particularly with VER and its derivatives, these stabilizing hydrogen bonds were consistently disrupted, leading to increased atomic fluctuations. This disruption reflects the allosteric perturbation of flap dynamics, which is a hallmark of competitive inhibition in BACE1. By destabilizing or displacing the flap loop, inhibitors prevent proper positioning of natural substrates, thereby impairing enzymatic turnover. Among the tested complexes, VERMOD-33 and VERMOD-57 displayed fluctuation patterns highly similar to the native VER-bound state. Both derivatives induced comparable levels of flexibility in the flap and catalytic loops, suggesting that they faithfully mimic the inhibitory mechanism of VER by locking the protein in a dynamic, substrate-inaccessible state. In contrast, VERMOD-9 produced a more pronounced increase in fluctuations, consistent with its higher RMSD and looser binding orientation, which may reflect a less efficient inhibitory effect. VERMOD-10 and VERMOD-168 induced moderate fluctuations, striking a balance between stability of the overall fold and perturbation of the catalytic environment.

Interestingly, the residues surrounding Tyr132, Thr133, and Gly134 within the flap loop exhibited the sharpest deviations, underscoring their role as hinge points during loop opening and closing motions. Similarly, residues around Asp289 and Thr292 in the catalytic loop showed elevated RMSF, further highlighting the sensitivity of the catalytic dyad region to ligand-induced perturbations. These local fluctuations are consistent with prior crystallographic and simulation studies, which established that BACE1 inhibitors often function by modulating loop plasticity rather than rigidly stabilizing the active site. Taken together, the RMSF analysis demonstrates that all derivatives maintain the essential inhibitory hallmark of VER, the induction of destabilizing fluctuations in the flap and catalytic loops. Among them, VERMOD-33 and VERMOD-57 emerge as the most faithful mimics of native VER dynamics, suggesting that they could preserve inhibitory efficacy while offering improved structural or pharmacological properties.

RoG and SASA analyses (Figure 6c,d) collectively confirmed that the global fold of BACE1 remained highly stable upon ligand binding. Across all complexes, the RoG fluctuated narrowly between 2.10 and 2.11 nm, with negligible deviations compared to the apo state. This observation indicates that neither VER nor its derivatives induced large-scale conformational rearrangements, unfolding, or collapse of the BACE1 architecture during the 200 ns trajectory. Instead, ligand binding primarily exerted localized effects within the binding cavity while preserving the overall tertiary structure of the protein. Similarly, the SASA values remained within a narrow range (176–179 nm^2^) across the systems, further corroborating the absence of global destabilization. Subtle differences were observed, however: VERMOD-168 consistently displayed the highest SASA (~179.22 nm^2^), suggesting that its bulkier scaffold and extended substituents slightly increased solvent exposure by expanding the binding cavity and contacting peripheral residues. This effect may be advantageous in terms of multi-sub-pocket engagement, though it could also lead to increased desolvation penalties during binding. In contrast, VER and VERMOD-33 maintained SASA values nearly identical to the apo state, reflecting compact binding without significantly perturbing solvent accessibility. The detailed RoG and SASA profiles for each complex are provided in Supplementary Data S4, with RoG plots shown in Supplementary Figure S1 and SASA plots in Supplementary Figure S2.

The ligand–protein center-of-mass distance analysis (Figure 6e) offered a more direct measure of binding persistence and positional stability within the active site. VER maintained a relatively tight distance (1.11 nm), consistent with its known inhibitory potency. Notably, VERMOD-33 exhibited an even closer and more stable distance (~0.99 nm), reinforcing its enhanced affinity and reduced drift within the catalytic pocket. By contrast, VERMOD-57 and VERMOD-9 showed larger average distances (1.38 and 1.37 nm, respectively), which is in agreement with their higher RMSD values and less stable orientations. These findings suggest that while both derivatives can occupy the binding site, their reduced positional stability weakens overall retention. VERMOD-10 and VERMOD-168 displayed intermediate values (~1.15–1.18 nm), indicating reasonably stable anchoring but not as tight as VER or VERMOD-33. Finally, the hydrogen bond occupancy profiles (Figure 6f) provided critical insights into the polar interaction landscape sustaining complex stability. VER maintained an average of ~3 hydrogen bonds throughout the trajectory, a pattern consistent with its crystallographic binding mode. Strikingly, VERMOD-33 achieved the highest occupancy (~4.3 bonds), reflecting additional or more persistent polar contacts that reinforce its superior stability compared to the parent compound. This enhancement aligns with its tighter center-of-mass distance and low RMSD, positioning VERMOD-33 as the most promising mimic of VER. On the other hand, VERMOD-57 and VERMOD-9 averaged ~2–3 bonds but with higher variability, consistent with their dynamic repositioning and weaker stabilization. VERMOD-10 was unique, forming the lowest number of persistent hydrogen bonds (~1 bond) despite being stable in overall conformation. This suggests that its stabilization may rely more on hydrophobic, π–π stacking, or electrostatic interactions rather than conventional hydrogen bonds. VERMOD-168 maintained ~3 hydrogen bonds on average, complementing its bulky scaffold and ability to engage multiple sub-pockets, which supports a balanced binding profile between stability and adaptability. These results demonstrate that ligand binding does not compromise BACE1’s global compactness or solvation but instead differentially tunes local retention, interaction networks, and active site flexibility. Among the tested derivatives, VERMOD-33 consistently outperformed others by maintaining close spatial retention, strong hydrogen bonding, and compact interaction profiles, highlighting its potential as a structurally optimized inhibitor with functional similarity to native VER.

### 2.6. MM/PBSA Free Energy Analysis and Per-Residue Decomposition

**Figure 7:**
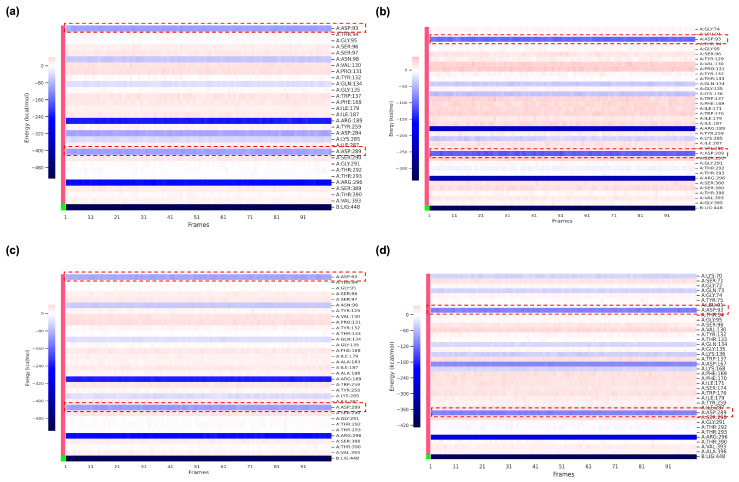
Heatmap of per-residue energy contributions (kcal/mol) in the ligand-binding domain of BACE1. (**a**) BACE1_VER complex. (**b**) BACE1_VERMOD-33 complex. (**c**) BACE1_VERMOD-57 complex. (**d**) BACE1_VERMOD-168 complex. Red dotted boxes represent the active binding residues of BACE1.

To further elucidate the thermodynamic determinants of ligand binding, MM/PBSA free energy calculations and per-residue decomposition analyses were performed for BACE1 in complex with VER and the top-performing derivatives (Table 6, Figure 7). The results clearly differentiated the binding strengths of the parent compound VER and its derivatives. Native VER exhibited a binding free energy of −35.33 ± 5.21 kcal/mol, consistent with its established inhibitory potency. Strikingly, several derivatives demonstrated more favorable energetics, with VERMOD-33 (−51.12 ± 4.99 kcal/mol) emerging as the most stable complex, followed by VERMOD-57 (−43.85 ± 4.42 kcal/mol) and VERMOD-168 (−37.55 ± 6.41 kcal/mol). These values significantly exceed the binding affinity of VER, underscoring the enhanced stabilizing features encoded in the modified scaffolds. By contrast, VERMOD-9 (−21.79 ± 5.31 kcal/mol) displayed weaker affinity, in line with its higher RMSD fluctuations and poorer retention within the binding pocket. VERMOD-10 (−33.77 ± 4.41 kcal/mol) was comparable to VER, suggesting a more conservative binding profile.

Per-residue energy decomposition (Figure 7) revealed the molecular origins of these differences. Across all ligand-bound systems, Asp93 and Asp289 consistently emerged as dominant contributors to binding stability. These residues form part of the catalytic dyad and flap loop environment, anchoring inhibitors through strong electrostatic and hydrogen bond interactions. In the VER complex (Figure 7a), both Asp93 and Asp289 provided favorable contributions, but the strength of these interactions was further amplified in complexes with the top-performing derivatives. For instance, VERMOD-33 (Figure 7b) showed markedly stronger stabilization energies at both Asp93 and Asp289 compared to VER, explaining its superior overall binding affinity. VERMOD-57 (Figure 7c) exhibited a similar pattern, with both residues contributing more favorably than in the VER system. In contrast, VERMOD-168 (Figure 7d) maintained moderate but consistent stabilization at these active-site residues, supporting its balanced multi-sub-pocket engagement. Importantly, the enhanced energetic contributions of Asp93 and Asp289 in derivative complexes align with the RMSF analysis, where these residues were also implicated in ligand-induced loop flexibility. This suggests a dual role: while ligand binding induces local fluctuations in these catalytic loops (antagonizing substrate accommodation), it simultaneously stabilizes direct inhibitor contacts through strong electrostatic anchoring. The result is a thermodynamically favorable but functionally inhibitory binding mode. These findings highlight the critical role of Asp93 and Asp289 as energetic hotspots for ligand recognition. The ability of derivatives such as VERMOD-33 and VERMOD-57 to exploit these residues more efficiently than native VER provides a compelling rationale for their enhanced binding affinities and inhibitory potential. This synergy between dynamic disruption of loop stability and strengthened energetic anchoring underscores the effectiveness of scaffold modifications in optimizing BACE1 inhibition.

### 2.7. In Silico Pharmacokinetics and ADMET Profiling of Verubecestat and Its Derivatives

To complement the structural and energetic analyses, the pharmacokinetic and ADMET properties of VER and its top-performing derivatives were evaluated using in silico tools (Table 7). This analysis provided insights into drug-likeness, absorption, metabolism, and toxicity liabilities, thereby helping to prioritize derivatives with the most favorable pharmacological profiles. The ADMET and pharmacokinetic profiling provided essential insights into the drug-likeness of VER and its top-performing derivatives. All compounds satisfied the basic drug-likeness requirements in terms of hydrogen bond donors (≤2) and acceptors (<10), suggesting good oral bioavailability potential according to Lipinski’s rules. The molecular weights of the derivatives were generally higher than VER, ranging from ~410 to ~499 g/mol, yet still within the acceptable threshold (<500 g/mol) for small-molecule drugs. Interestingly, VERMOD-33 and VERMOD-57 retained relatively modest molecular weights (410.835 and 414.403 g/mol, respectively), making them attractive candidates for further optimization.

The lipophilicity profile, as indicated by cLogP, highlighted notable differences among the analogs. VER exhibited a low cLogP (0.483), suggesting poor membrane permeability, which is consistent with its limited oral absorption (13.9%). By contrast, VERMOD-33 displayed a markedly higher cLogP (3.482), correlating with improved membrane permeability and the highest predicted human oral absorption (29.1%). VERMOD-57 showed moderate lipophilicity (1.321) and also achieved relatively high oral absorption (27.4%), striking a favorable balance between solubility and permeability. In contrast, highly polar analogs such as VERMOD-10 (PSA 159.15 Å^2^) and VERMOD-9 (PSA 145.21 Å^2^) exhibited reduced lipophilicity and correspondingly lower oral absorption values (17.1% and 15.6%, respectively), reflecting the impact of polar surface area on gastrointestinal absorption. The pharmacokinetic descriptors also provided insights into distribution and metabolism. The predicted BBB penetration (QPlogBB) values indicated that VERMOD-33 (0.273) has the highest potential for CNS access, which is desirable for BACE1 inhibition in Alzheimer’s disease therapy. In contrast, VERMOD-10 and VERMOD-9 displayed negative QPlogBB values (−1.051 and −0.799), suggesting poor CNS permeability and limiting their potential as effective brain-targeting drugs. Consistent with these findings, the Caco-2 permeability (QPPCaco) and MDCK cell permeability (QPPMDCK) parameters further supported VERMOD-33’s superior absorption and distribution potential, as it recorded the highest values in both assays (9.576 and 13.029, respectively).

From a toxicity standpoint, none of the derivatives exhibited mutagenic, tumorigenic, reproductive, or irritant properties, underscoring their safety potential in early-stage screening. However, QPlogHERG values for all compounds were in the range of −5.3 to −7.5, indicating possible hERG channel inhibition risk, a common liability for CNS-active drugs. Among them, VERMOD-33 showed a moderate risk (−5.699), whereas VERMOD-57 and VERMOD-9 (−7.134 and −7.480, respectively) presented slightly higher risks that warrant careful optimization in further stages of drug development. Overall, the ADMET profiling highlighted VERMOD-33 as the most promising derivative, combining favorable lipophilicity, superior oral absorption, enhanced BBB penetration, and strong permeability across predictive models, all while maintaining an acceptable safety profile. VERMOD-57 also emerged as a strong candidate, balancing potency with reasonable absorption and distribution parameters, although its predicted CNS exposure is less favorable than VERMOD-33. In contrast, VERMOD-9 and VERMOD-10, despite demonstrating binding activity, face limitations in pharmacokinetics due to their high polarity and poor predicted CNS penetration. VERMOD-168, with the highest molecular weight (499.569 g/mol) and surface area, showed moderate absorption and distribution values, reflecting a bulkier scaffold that may require optimization for CNS delivery.

### 2.8. Results of Retrosynthetic Design for Verubecestat Derivatives

**Figure 8:**
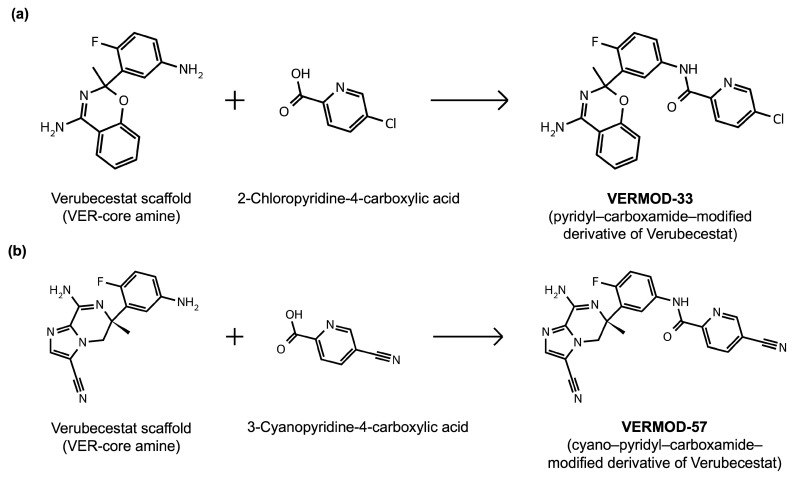
Retrosynthetic routes toward prioritized Verubecestat (VER) derivatives. (**a**) Retrosynthetic pathway leading to VERMOD-33, reconstructed from the VER-core amine fragment and 2-chloropyridine-4-carboxylic acid via amide bond formation. (**b**) Retrosynthetic pathway leading to VERMOD-57, obtained from the VER-core amine fragment and 3-cyanopyridine-4-carboxylic acid through a similar amide coupling strategy.

Rational optimization of lead scaffolds requires not only structural and energetic analyses but also a clear understanding of how the proposed derivatives could be synthesized in practice. To address this, retrosynthetic analysis was performed for two of the top-performing derivatives, VERMOD-33 and VERMOD-57, using computer-aided design tools. This approach enables the dissection of complex drug-like molecules into synthetically accessible building blocks, guiding medicinal chemists toward practical routes for compound preparation. Figure 8 illustrates the representative retrosynthetic pathways and precursor fragments for these derivatives. The first route focuses on VERMOD-33, a derivative characterized by the incorporation of a 2-chloropyridine-4-carboxamide moiety into the parent VER scaffold. Retrosynthetic disconnection breaks this compound down into two key precursors: (i) the Verubecestat scaffold bearing a reactive amine group and (ii) 2-chloropyridine-4-carboxylic acid, a commercially available heteroaryl acid. The coupling of these two fragments via amide bond formation reconstructs the final product. This strategy exploits a well-established synthetic transformation and is therefore highly accessible from a laboratory standpoint. From a computational perspective, the retrosynthetic evaluation of this pathway produced a precursor score of −320 and an average model score of 0.59, both supportive of synthetic feasibility. The plausibility score of 1.00 further underscores the robustness of this route. Importantly, the introduction of the chloropyridine ring is not only synthetically feasible but also pharmacologically meaningful: the chloro substituent may enhance lipophilic interactions within hydrophobic subpockets of BACE1, while the pyridine nitrogen provides additional opportunities for hydrogen bonding. Thus, the retrosynthetic design of VERMOD-33 highlights a convergence of chemical accessibility and pharmacological rationality.

The second pathway describes the synthesis of VERMOD-57, a derivative modified by the introduction of a 3-cyanopyridine-4-carboxamide group. Retrosynthetic disconnection reveals two analogous precursors to those used in the first pathway: (i) the Verubecestat scaffold with a free amine functionality and (ii) 3-cyanopyridine-4-carboxylic acid, an electron-deficient heteroaryl acid. The assembly of VERMOD-57 proceeds via a similar amide coupling reaction, linking the amine of the VER core to the carboxyl group of the cyanopyridine derivative. Interestingly, this pathway received a precursor score of −460 and an average model score of 0.45, slightly lower than that of VERMOD-33. While the plausibility score remained at 1.00, suggesting feasibility, the reduced model score reflects the additional synthetic challenge posed by the electron-withdrawing cyano group, which may influence reaction efficiency and product yield. Nevertheless, the incorporation of the cyano functionality offers potential pharmacological advantages: it increases polarity, expands hydrogen-bonding potential, and may contribute to stronger anchoring within the catalytic pocket of BACE1. Thus, although VERMOD-57 may be synthetically more demanding than VERMOD-33, its structural features justify further exploration. The retrosynthetic designs of VERMOD-33 and VERMOD-57 demonstrate that selective amide couplings with heteroaryl carboxylic acids provide a modular and practical route to scaffold diversification. This modularity is particularly valuable in medicinal chemistry campaigns, as it allows rapid generation of derivative libraries with varying electronic, steric, and hydrogen-bonding properties. The computational scoring further indicates that VERMOD-33 is synthetically more accessible, while VERMOD-57, though less straightforward, introduces favorable pharmacological elements.

## 3. Discussion

This work implemented a comprehensive in silico workflow to revisit the therapeutic potential of VER, a potent but clinically unsuccessful BACE1 inhibitor, and to design next-generation derivatives with improved profiles. VER was one of the most advanced BACE1 inhibitors in clinical development, yet its failure in Phase III Alzheimer’s trials highlighted significant challenges in balancing potency, CNS penetration, and safety [20,21]. Our integrated approach, spanning molecular docking, FMO analysis, pharmacophore modeling, MD simulation, MM/PBSA free energy decomposition, retrosynthetic analysis, and in silico ADMET profiling, enabled a systematic evaluation of VER and rationally designed derivatives. The results converge on two optimized scaffolds, VERMOD-33 and VERMOD-57, which demonstrate superior binding affinity, consistent inhibitory dynamics, and more favorable pharmacokinetics compared to the parent compound. Docking and pharmacophore mapping successfully recapitulated the canonical BACE1 binding architecture of VER, where anchoring at the catalytic dyad (Asp93 and Asp289) and stabilization via flap-region residues are indispensable for inhibitory activity [22]. Importantly, VERMOD-33 and VERMOD-57 preserved this dyad-centered hydrogen-bonding network while extending deeper into hydrophobic pockets surrounding the S2’ and flap regions. This modification increased van der Waals complementarity and shape fit, thereby enhancing predicted affinity. Such optimization strategies are well supported by previous SAR studies, which show that hydrophobic pocket extensions can significantly improve both potency and selectivity of BACE1 inhibitors [23,24]. In contrast, derivatives such as VERMOD-9 and VERMOD-10 displayed misaligned binding poses and reduced complementarity, consistent with their higher RMSDs and weaker energy scores.

The MD simulations provided further mechanistic clarity. The RMSF profiles revealed that all inhibitors induced elevated flexibility in the flap and catalytic dyad loops (Leu91–Gly135 and Asp289–Ala305), regions crucial for substrate accommodation. This loop destabilization is a hallmark of inhibitory binding, consistent with earlier computational and experimental studies indicating that productive inhibitors interfere with flap rigidity and prevent substrate access [25,26]. Among the derivatives, VERMOD-33 and VERMOD-57 most closely mirrored the dynamic signature of native VER, but with enhanced persistence of polar contacts and reduced ligand drift. In contrast, VERMOD-9 and VERMOD-10 failed to maintain stable interactions, displaying higher center-of-mass distances and fluctuating hydrogen-bond occupancies. Notably, VERMOD-33 achieved the highest hydrogen-bond occupancy (~4 bonds), exceeding that of VER (~3 bonds), underscoring its superior stabilizing effect. The MM/PBSA free energy analysis corroborated these findings, with VERMOD-33 exhibiting the most favorable ΔG_binding (≈−51.1 kcal/mol), substantially surpassing VER (≈−35.3 kcal/mol). Per-residue decomposition pinpointed Asp93 and Asp289, and nearby flap residues as dominant contributors to ligand stabilization, residues that have been repeatedly validated as hot spots for inhibitor design [22]. VERMOD-33 and VERMOD-57 engaged these residues more strongly than VER, reflecting their optimized scaffolds. These findings align with fragment-based and macrocyclic inhibitor designs that emphasize multi-residue engagement across both polar and hydrophobic sub-sites to maximize binding affinity [27,28,29].

ADMET predictions shed light on why VER ultimately failed in the clinic and how derivative optimization may address these shortcomings. VER exhibited low predicted oral absorption (13.9%), moderate BBB penetration (QPlogBB ≈ −0.23), and a concerning risk of hERG inhibition (QPlogHERG ≈ −5.36), which resonates with clinical observations of poor efficacy and potential off-target liabilities [21,30]. In contrast, VERMOD-33 showed markedly improved oral absorption (29%), favorable BBB penetration (QPlogBB ≈ 0.27), and maintained a clean toxicity profile, suggesting an improved pharmacological balance. VERMOD-57 also improved pharmacokinetic parameters but displayed somewhat reduced BBB penetration compared to VERMOD-33. Interestingly, VERMOD-168, while demonstrating strong multi-pocket interactions, suffered from excessive molecular weight and surface area, limiting its predicted CNS availability. This trade-off highlights a key challenge in BACE1 inhibitor optimization: striking a balance between potency-enhancing scaffold modifications and CNS drug-likeness [7,31,32]. From a translational perspective, these findings emphasize that VER’s failure does not necessarily invalidate the therapeutic value of BACE1 inhibition, but rather underscores the need for carefully tuned scaffolds. Previous large-scale trials with BACE1 inhibitors, including lanabecestat, atabecestat, and elenbecestat, consistently showed that off-target effects, suboptimal CNS penetration, and excessive amyloid-lowering contributed to poor outcomes [11,15,20]. The derivatives identified here, especially VERMOD-33, appear to address several of these issues by enhancing binding while maintaining physicochemical properties compatible with CNS penetration. Moreover, the detailed residue-level insights may support hybrid strategies such as dual-target inhibitors (BACE1–GSK3β or BACE1–γ-secretase modulators) that address the multifactorial nature of Alzheimer’s pathology [33,34,35].

## 4. Materials and Methods

### 4.1. Three-Dimensional Structure Construction, Rational Modifications, and MM2 Energy Minimization

The three-dimensional structure of the parent compound VER was constructed using Chem3D Ultra 22.0 (PerkinElmer, Cambridge, MA, USA). VER is a potent, selective, and structurally unique BACE1 inhibitor. However, its clinical failure underscores the need for rational redesign to overcome limited efficacy and safety concerns. In this study, we employed an integrative molecular modeling strategy to generate an extensive derivative library aimed at rescuing VER’s therapeutic potential. The initial geometry of VER was optimized using the MM2 force field, which ensures physically realistic bond lengths, bond angles, torsional strain, and steric packing. The same protocol was applied consistently to all subsequent derivatives to standardize the conformational landscape prior to computational evaluations. Rational modifications were introduced around the VER scaffold with the goal of improving binding affinity, metabolic stability, blood–brain barrier penetration, and selectivity toward BACE1. The modifications were conceptually grouped into six broad categories, including alkoxy substitutions, alkyl substitutions, bioisosteric replacements, carbonyl swaps, halogen substitutions, and halogenated heterocycles. Rather than focusing narrowly on single-point changes, our strategy explored chemical space across both proximal and distal regions of the scaffold, thereby enabling the identification of novel binding vectors. The alkoxy series introduced diverse electron-donating or sterically modulated substituents such as methoxy, ethoxy, propoxy, and fluorinated alkoxy groups, while the alkyl series incorporated small to bulky groups such as methyl, difluoromethyl, trifluoromethyl, and dimethylaminomethyl moieties to probe hydrophobic interactions within the BACE1 active site.

Bioisosteric replacements represented a major axis of exploration, wherein classical pharmacophoric elements were replaced by heteroaryl motifs such as thiazole, oxadiazole, tetrazole, and isoxazoline, alongside carbamate- and oxime-like fragments. These replacements were designed to refine hydrogen-bonding interactions, modify electronic properties, and potentially reduce off-target effects. Carbonyl-centered modifications, including amide-to-thioamide swaps and related transformations, further probed the electronic flexibility of the scaffold while tuning its hydrogen-bonding capacity. In parallel, halogen substitutions were systematically introduced at strategic positions across aromatic and heteroaromatic regions, with fluorine, chlorine, bromine, and iodine substitutions tested individually and in combination to optimize π-stacking, dipole modulation, and metabolic resilience. Finally, halogenated heterocycles and cyano-substituted rings were incorporated to provide conformational rigidity and expand the repertoire of potential pharmacophoric orientations within the binding pocket. Through this comprehensive structural engineering framework, a total of 200 VER derivatives (VERMOD-1 to VERMOD-200) were generated. Each derivative was energy-minimized using MM2 to achieve stable, low-energy conformations suitable for downstream computational analyses. This systematic approach not only ensured chemical feasibility but also created a chemically diverse derivative library designed to explore the full spectrum of BACE1 binding dynamics and therapeutic optimization.

### 4.2. Three-Dimensional Structure Alignment and Similarity Analysis

To systematically evaluate structural consistency and conformational diversity among VER and its 200 rationally designed derivatives, we employed a two-step similarity assessment that combined three-dimensional alignment and molecular fingerprint–based comparisons. Conformational alignment was performed using the superimpose tool in BIOVIA Discovery Studio 2024 (Dassault Systèmes, Vélizy-Villacoublay, France) [36], enabling visualization of atomic overlap, scaffold preservation, and pharmacophoric feature distribution across the derivative library. This facilitated direct inspection of structural modifications while ensuring that essential backbone features of VER were retained. To complement this qualitative alignment, quantitative similarity scores were computed using the Tanimoto coefficient, derived from cheminformatics fingerprints. Two fingerprinting strategies were adopted: (i) FP2 (path-based fingerprints), which capture linear atom paths up to a defined bond length [37,38], and (ii) Morgan fingerprints (ECFP4), which encode circular atom environments and provide enhanced sensitivity to local substitutions [19,39]. These fingerprints were generated using the RDKit toolkit (Python: https://www.rdkit.org/) (accessed 14 May 2025) [40], with custom scripts developed to perform pairwise similarity calculations, produce bar chart rankings of each derivative against native VER, and generate hierarchical clustering heatmaps to visualize overall structural relationships. This integrative analysis established a robust framework for quantifying structural conservation and divergence across the VER derivative library. By mapping similarity trends, we were able to identify subsets of derivatives that preserved core pharmacophoric features while introducing meaningful chemical diversity, thereby prioritizing them for subsequent molecular docking, MD simulations, and free-energy profiling.

### 4.3. Molecular Docking Simulations and Binding Affinity Analysis

Molecular docking was performed to characterize the interactions between VER, its structural derivatives, and the BACE1. The primary goal was to identify key amino acid residues mediating ligand–protein binding, define the types of intermolecular forces contributing to affinity, and compare binding orientations across the designed derivatives to evaluate how specific modifications influence binding performance. The crystal structure of BACE1 (PDB ID: 5HTZ [22], chain A; resolution 1.95 Å) was retrieved from the RCSB Protein Data Bank. This structure contains the native ligand (VER) which was used for docking validation. Prior to docking, the receptor structure was refined using Swiss-PdbViewer v4.1 (Swiss Institute of Bioinformatics, Lausanne, Switzerland) [41], which involved removing crystallographic water molecules, performing energy minimization, and repairing missing side chains to yield an optimized structure suitable for ligand docking. The active site and binding pockets were annotated using PDBSum (European Bioinformatics Institute, Cambridge, UK) [42], ensuring accurate localization of functionally relevant residues. To validate the docking protocol, the native ligand VER was redocked into the BACE1 catalytic pocket using identical parameters. The resulting docked pose reproduced the experimental conformation with an RMSD of ≤2.0 Å, confirming the reliability and accuracy of the docking setup.

Molecular docking simulations were carried out using the HADDOCK v2.4 platform (University of Utrecht, Utrecht, Netherlands), which differs from conventional docking tools by integrating ambiguous interaction restraints (AIRs) derived from experimental or computational data, thereby enhancing prediction accuracy and biological relevance [43,44]. The standalone interface enabled advanced customization of docking parameters, including both geometric and energetic constraints, to improve reliability. Docking outputs were evaluated through cluster analysis and HADDOCK scoring functions. Binding models were grouped into clusters, with the largest cluster considered the most representative due to its reproducibility and stability. The HADDOCK score, a composite of van der Waals, electrostatics, desolvation energy, and buried surface area contributions, was used to rank docking poses. The top-ranked complex with the most favorable score was selected as the representative binding model. To complement docking outcomes, binding free energies (ΔG, kcal/mol) were estimated using the PRODIGY-LIG web server (https://rascar.science.uu.nl/prodigy/lig) (accessed 14 May 2025), which calculates ΔG based on structural determinants such as intermolecular contacts, buried surface area, and desolvation effects. The methodology follows the framework described by Vagone, A., and Bonvin, AM [45]. This combined strategy provided both qualitative insight into binding modes and quantitative predictions of ligand–protein affinity, enabling robust comparison of VER derivatives and identification of promising candidates for further study.

### 4.4. HOMO–LUMO Analysis of Verubecestat Derivatives

The frontier molecular orbital (FMO) characteristics of VER and its designed derivatives were investigated using density functional theory (DFT) implemented through a custom Python workflow [46]. All molecular structures were fully optimized at the PBE0/6-31G(d) level of theory, which offers a balance between computational efficiency and reliable electronic structure prediction [47]. Convergence criteria were set to tight self-consistent field (SCF) thresholds, with an energy tolerance of 10^−6^ Ha and a maximum gradient of 10^−3^ Ha/Bohr, ensuring accurate and stable optimization [48]. Following geometry optimization, single-point energy calculations were conducted at the same level to obtain orbital eigenvalues. The script automatically identified the highest occupied molecular orbital (HOMO) and lowest unoccupied molecular orbital (LUMO) and computed the energy gap (ΔE = E_LUMO − E_HOMO). This parameter is a critical descriptor of electronic excitation potential, molecular stability, and chemical reactivity [49]. To visualize orbital delocalization, frontier orbital iso-surfaces were generated using standard visualization tools, with an isovalue of 0.02 a.u. [50,51], enabling assessment of how specific structural modifications affect electron density distribution across the molecular framework. This computational protocol provided a consistent and reproducible framework for comparing the electronic properties of VER derivatives, thereby supporting rationalization of their reactivity and potential activity as novel BACE1 inhibitors.

### 4.5. Three-Dimensional Pharmacophore Modeling

Three-dimensional (3D) pharmacophore modeling was performed to elucidate the key molecular features underlying the interactions of VER and its derivatives with BACE1. This strategy provides a structural basis for understanding how distinct chemical functionalities contribute to receptor recognition and ligand affinity. Pharmacophore models were generated using LigandScout version 4.5 (Inte:Ligand, Vienna, Austria) [52], which identifies and maps critical interaction features such as hydrogen bond donors (HBDs), hydrogen bond acceptors (HBAs), hydrophobic moieties, and electrostatic interaction centers. These elements represent the fundamental determinants of ligand–protein binding specificity and stability. By employing LigandScout’s automated feature detection and alignment algorithms, comparative pharmacophore models were constructed for VER and its derivatives. This analysis facilitated the identification of conserved interaction motifs and highlighted structural modifications that either preserved or enhanced key pharmacophoric features, providing insights into the molecular basis of binding optimization.

### 4.6. Molecular Dynamics (MD) Simulation for Structural Stability and Interaction Analysis

The MD simulations were conducted to explore the conformational dynamics, structural stability, and interaction patterns of VER and its derivatives in complex with BACE1. All simulations were performed using GROMACS version 2025.2 [53]. Ligand topologies were generated with the General Amber Force Field (GAFF2), and atomic partial charges were assigned via the AM1-BCC method [54,55] using acpype integrated with AmberTools21. The receptor was parameterized with the AMBER99SB force field, ensuring consistency in treating bonded and non-bonded interactions. The protein–ligand complexes were solvated in a dodecahedral box with the SPC water model, maintaining a minimum distance of 1.4 nm between solute atoms and box boundaries. Periodic boundary conditions (PBC) were applied in all directions, and the systems were neutralized with counterions, followed by the addition of 100 mM NaCl to mimic physiological ionic strength. Non-bonded interactions were handled with a 1.2 nm cutoff for van der Waals forces, while long-range electrostatics were calculated with the Particle Mesh Ewald (PME) method [56]. Energy minimization was performed using the steepest descent algorithm until the maximum force was below 1000 kJ/mol/nm, removing steric clashes and optimizing the starting structure. Equilibration was carried out in two steps: (i) NVT ensemble for 100 ps, applying positional restraints to stabilize temperature at 310 K using the Berendsen thermostat, followed by (ii) NPT ensemble for 100 ps, equilibrating pressure at 1 bar with the Berendsen barostat. Subsequently, unrestrained 200 ns production runs were performed, with temperature maintained using the V-rescale thermostat and pressure regulated via the Parrinello–Rahman barostat. Trajectories were recorded every two fs for post-simulation analyses. Trajectory processing and structural evaluation focused on monitoring RMSD, RMSF, RoG, SASA, hydrogen bonding patterns, and binding pocket dynamics. Interaction profiles were analyzed using PyMOL version 3.1.3 (Schrödinger LLC, New York, NY, USA) [57], BIOVIA Discovery Studio 2024 (Dassault Systèmes, San Diego, CA, USA) [36], and UCSF ChimeraX version 1.10.1 (University of California, San Francisco, CA, USA) [58], enabling both quantitative measurements and qualitative visualization of the ligand–protein dynamics.

### 4.7. Molecular Mechanics/Poisson–Boltzmann Surface Area (MM/PBSA) Calculations

The binding free energies of VER and its derivatives in complex with BACE1 were estimated using the MM/PBSA method, applied to equilibrated frames extracted from the MD trajectories. This approach integrates gas-phase molecular mechanics energies, solvation effects, and entropic contributions to provide a comprehensive evaluation of ligand–protein binding affinity [59]. For each snapshot, three principal energetic terms were calculated: (i) molecular mechanics energy in the gas phase (electrostatic and van der Waals interactions), (ii) solvation free energy, composed of a polar component derived from the Poisson–Boltzmann continuum model and a nonpolar component estimated from solvent-accessible surface area (SASA), and (iii) entropic contributions accounting for the conformational flexibility of both ligand and receptor. The Single-Trajectory Protocol (STP) was adopted, whereby the receptor, ligand, and complex free energies were computed from the same MD trajectory. This strategy reduces computational cost and improves consistency by assuming limited conformational rearrangements upon binding [60,61,62]. Free energy calculations were carried out using gmx_MMPBSA version 1.6.4, integrated with the GROMACS simulation framework [63]. The overall binding free energy (ΔG_binding) was determined according to the standard thermodynamic cycle:

Δ*G_binding* = Δ*G_complex* − Δ*G_ligand* − Δ*G_receptor*

where ΔG_complex is the total free energy of the solvated protein–ligand complex, ΔG_ligand represents the free energy of the unbound ligand in solution, and ΔG_receptor denotes the free energy of the isolated receptor. The calculated ΔG_binding values provided a quantitative basis for comparing derivatives and identifying modifications that confer improved binding affinity and interaction stability relative to native VER.

### 4.8. In Silico Pharmacokinetics and ADMET Evaluation

The pharmacokinetic profiles and drug-likeness of VER derivatives were evaluated using a multi-platform computational strategy. SwissADME (Swiss Institute of Bioinformatics, Lausanne, Switzerland) [64] was first applied to calculate key physicochemical descriptors, including molecular weight, lipophilicity (MlogP), HBD, HBA, and topological polar surface area (TPSA). These parameters were compared against Lipinski’s Rule of Five, a benchmark for estimating oral bioavailability. Derivatives meeting these criteria were considered more likely to demonstrate favorable absorption and permeability, supporting their potential as orally active drugs. To complement these assessments, DataWarrior version 6.4.1 (OpenMolecules, Karlsruhe, Germany) [65] was used to evaluate drug-likeness and toxicity risk. This tool integrates molecular descriptors with predictive models to identify potential toxicological concerns, including mutagenicity, tumorigenicity, reproductive toxicity, and irritant properties, thereby flagging structural liabilities at an early stage. A more detailed pharmacokinetic and ADMET profiling was performed using QikProp (Schrödinger, New York, NY, USA) [66]. QikProp computed advanced descriptors related to absorption, distribution, metabolism, and excretion (ADME), including solvent-accessible surface area (SASA), hydrophobic SASA (FOSA), hydrophilic SASA (FISA), percentage of predicted human oral absorption, QPlogHERG (hERG channel inhibition, a measure of cardiotoxicity), QPPCaco (Caco-2 cell permeability), QPlogBB (blood–brain barrier penetration), QPPMDCK (MDCK cell permeability), QPlogKp (skin permeability), and QPlogKhsa (serum protein binding). Collectively, these evaluations provided a robust computational framework for prioritizing derivatives with optimized pharmacokinetic behavior, reduced toxicity risks, and improved drug-likeness relative to VER.

### 4.9. Retrosynthetic Design of Verubecestat Derivatives

Retrosynthetic analysis of VER and its potential derivatives was performed using the ASKCOS platform (https://askcos.mit.edu/) (accessed 14 May 2025) [67]. The retrosynthesis engine was configured with a Relevance Heuristic precursor scoring system to prioritize chemically meaningful starting materials, while atom mapping was carried out using RXNMapper version 0.4.2 [68]. To ensure practical feasibility, a minimum plausibility threshold of 0.001 was applied, with regioselectivity checking and near-cycle filtering enabled to avoid unrealistic disconnections. The retrosynthetic search was limited to the top 5 candidate results per iteration, balancing computational efficiency and chemical diversity. For pathway generation, Strategy 1 was employed using the template_relevance model with the Reaxys template set, capped at a maximum of 1000 reaction templates and a cumulative probability cutoff of 0.999.

## 5. Limitations and Future Works

Despite the encouraging findings from this study, several limitations should be acknowledged. First, although molecular docking, pharmacophore modeling, MD simulations, and MM/PBSA free energy calculations provided valuable insights into the stability and energetics of VER and its derivatives, these methods primarily assess noncovalent interactions within the BACE1 catalytic pocket. In reality, BACE1 inhibition involves a complex interplay of dynamic active-site rearrangements and multipoint hydrogen bonding with the catalytic aspartates (Asp93 and Asp289), which may not be fully captured within the timescales and resolution of classical MD simulations. Furthermore, long-range allosteric contributions or transient loop conformations that influence inhibitor binding were not explicitly addressed. Future work employing enhanced sampling techniques such as accelerated MD or meta-dynamics could provide a more comprehensive picture of the conformational landscape of BACE1 and the adaptive behavior of novel VER derivatives. Second, while the pharmacophore modeling successfully identified key interaction features consistent with the docking results, a direct quantitative comparison between pharmacophore-screened hits and their corresponding docked poses was not performed. This limitation stems from the current computational scope and the capabilities of the employed pharmacophore tools, which were primarily optimized for feature generation rather than pose correlation. Future studies should integrate pharmacophore–docking cross-validation workflows to further enhance methodological robustness and consistency. Third, the current framework did not explicitly model potential off-target interactions. BACE1 shares structural similarities with other aspartyl proteases, including BACE2 and cathepsin D, raising the possibility of cross-reactivity that can drive off-target toxicity. While in silico selectivity profiling can provide preliminary estimates, the absence of direct modeling against these homologs limits the current conclusions. A more robust strategy would involve systematic docking and MD simulations across a panel of related proteases, followed by free-energy perturbation (FEP) methods to quantify differential binding affinities. Such comparative modeling would be essential in establishing the therapeutic window of novel VER derivatives and minimizing off-target liabilities.

Although ADMET predictions suggested that the prioritized VERMOD analogs (e.g., VERMOD-33 and VERMOD-57) may exhibit improved BBB permeability, oral absorption, and reduced toxicity alerts compared with parent VER, these remain computational approximations. Critical parameters such as metabolic stability, efflux liability via P-glycoprotein, and long-term safety cannot be fully captured through predictive models alone. Experimental validation through in vitro enzymatic assays, hepatocyte clearance studies, and blood–brain barrier permeability assays (e.g., PAMPA-BBB, MDCK-MDR1) will be indispensable to corroborate these predictions. Moreover, the clinical failure of VER was partly attributed to adverse cognitive effects possibly linked to BACE1’s physiological roles in synaptic function. Therefore, functional neurotoxicity assays and behavioral readouts in preclinical models should be integrated into the following stages of evaluation. Finally, while this study demonstrates the feasibility of rescuing VER via rational molecular design, translation toward therapeutically viable candidates requires further experimental investment. Future work should prioritize three directions: (i) chemical synthesis of the most promising VERMOD analogs, followed by biochemical assays to confirm binding affinity and inhibition kinetics against BACE1; (ii) in vitro ADMET and toxicity profiling to establish pharmacokinetic and safety parameters; and (iii) extended computational pipelines incorporating longer simulation timescales, covalent docking methods (given VER’s potential electrophilic liabilities), and hybrid QM/MM simulations to capture proton transfer events in the catalytic dyad. Such integrative efforts will not only validate the computational findings but also help refine SAR principles guiding next-generation BACE1 inhibitor design.

## 6. Conclusions

In conclusion, this study employed an integrative computational framework to systematically assess VER and its rationally designed derivatives as BACE1 inhibitors. Our findings demonstrate that while native VER maintains moderate binding affinity and stability, several designed derivatives (most notably VERMOD-33 and VERMOD-57) exhibited superior binding free energies, enhanced pharmacophore complementarity, and improved interaction persistence within the BACE1 catalytic pocket. These derivatives effectively mimicked the inhibitory mechanism of VER while extending interactions into adjacent sub-pockets, providing a more favorable energetic and structural profile. Dynamic simulations highlighted that VER derivatives consistently induced increased flexibility in the loop regions flanking the catalytic dyad (Asp93 and Asp289), a mechanistic hallmark of inhibitory activity. In particular, VERMOD-33 and VERMOD-57 displayed fluctuation patterns that closely mirrored those of native VER, reinforcing their potential to act as next-generation inhibitors by destabilizing substrate-accommodating loops. Importantly, ADMET and pharmacokinetic predictions suggested that the prioritized derivatives retained favorable drug-like properties, with improved absorption potential and blood–brain barrier permeability compared to native VER, while showing no major mutagenic, tumorigenic, or reproductive toxicity alerts. These results highlight that carefully designed modifications of the VER scaffold can overcome some of the pharmacological shortcomings that hindered its clinical success, paving the way for rational re-engineering of BACE1 inhibitors. Overall, this work establishes a strong computational foundation for the optimization and rescue of VER-based scaffolds. By integrating structural, energetic, and pharmacokinetic perspectives, the study provides a roadmap for prioritizing candidate molecules for synthesis and preclinical validation. While experimental studies will be essential to confirm these predictions, the present framework underscores the potential of in silico design to revive clinically challenged drug scaffolds and advance next-generation therapeutics for Alzheimer’s disease.

## Figures and Tables

**Table 1:** Representative structural modifications of Verubecestat (VER) derivatives showing Tanimoto similarity scores, SMILES notation, and 2D representations. The derivatives listed here were selected based on interaction similarity to native VER. The complete set of 200 designed derivatives is provided in Supplementary Data S1.

Molecule	Modification Category	Tanimoto Similarity (FP2)	SMILES	2D Structure
VER	N/A	1.00	C[C@]1(CS(=O)(=O)N(C(=N1)N)C)C2=C(C=CC(=C2)NC(=O)C3=NC=C(C=C3)F)F	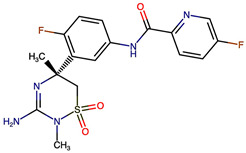
VERMOD-10	Alkoxy Substitution	0.69	CN1C(N)=N[C@@]2(C)[C@H](CCOC3=C2C=C(NC(=O)C2=NC=C(C=C2)C#N)C=C3)S1(=O)=O	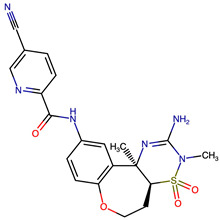
VERMOD-33	Alkyl Substitution	0.56	CC1(OC2=C(C=CC=C2)C(N)=N1)C1=CC(NC(=O)C2=CC=C(Cl)C=N2)=CC=C1F	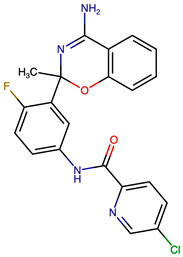
VERMOD-36	Bioisosteric Replacement	0.67	CN=[S@@]1(=O)C[C@](C)(NC(=N)N1C)C1=C(F)C=CC(NC(=O)C2=NC=C(Cl)C=C2)=C1	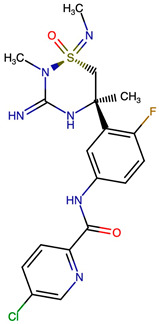
VERMOD-57	Carbonyl Swap	0.59	C[C@]1(CN2C(=CN=C2C(N)=N1)C#N)C1=C(F)C=CC(NC(=O)C2=NC=C(C=C2)C#N)=C1	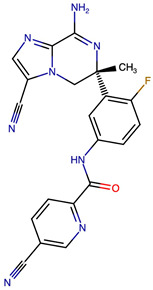
VERMOD-72	Halogen Substitution	0.78	COC1=CN=C(C=N1)C(=S)NC1=CC(F)=C(F)C(=C1)[C@]1(C)CS(=O)(=O)N(C)C(N)=N1	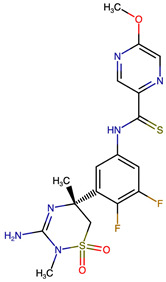
VERMOD-94	Halogenated Heterocycle	0.57	NC1=N[C@@](CF)([C@H]2C[C@H]2O1)C1=CC(NC(=O)C2=NC=C(C=C2Cl)C#N)=CC=C1F	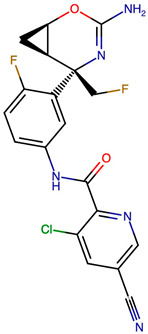
VERMOD-168	Ring Modification	0.70	CC#CCOC1=CN=C(C=N1)C(=O)NC1=CC(=C(F)C=C1)[C@]1(C)C[S@@]2(=O)=NCCCCN2C(N)=N1	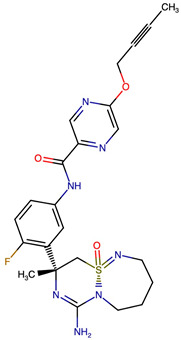

**Table 2:** Comparative docking results of Verubecestat (VER) and top-performing derivatives with BACE1, including binding affinities and interaction energy components. The complete molecular docking results are presented in Supplementary Data S2.

Complex	HADDOCK Score (a.u.)	Binding Energy (kcal/mol)	Van der Waals Energy	Electrostatic Energy	Desolvation Energy	RMSD	Hydrogen Bonds
BACE1_VER	−44.1 ± 0.5	−8.44	−29.1 ± 0.6	−133.4 ± 1.6	−1.7 ± 0.4	0.1 ± 0.1	Asp93, Pro131, Thr133, Asp289
BACE1_VERMOD-33	−53.9 ± 0.9	−10.42	−30.0 ± 0.8	−197.8 ± 8.2	−5.0 ± 0.4	0.1 ± 0.0	Asp93, Pro131, Lys168, Asp289
BACE1_VERMOD-57	−62.2 ± 0.7	−10.11	−36.9 ± 0.3	−240.0 ± 10.5	−1.4 ± 0.4	0.1 ± 0.0	Gln73, Asp93, Pro131, Asp289, Arg296
BACE1_VERMOD-9	−54.9 ± 0.5	−9.79	−31.5 ± 0.8	−216.5 ± 24.7	−2.6 ± 1.6	0.1 ± 0.1	Asp93, Pro131, Lys285, Asp289
BACE1_VERMOD-10	−52.7 ± 2.2	−9.79	−33.2 ± 1.0	−202.6 ± 3.5	−1.0 ± 0.5	0.1 ± 0.0	Asp93, Ser97, Thr133, Arg189, Tyr259, Asp289
BACE1_VERMOD-168	−51.8 ± 1.2	−9.05	−37.8 ± 0.5	−122.7 ± 10.3	−3.5 ± 0.3	0.1 ± 0.0	Gln73, Asp93, Thr133, Asp289, Thr293

**Table 3:** Molecular interaction profiles of BACE1 with Verubecestat (VER) and top-performing derivatives. The complete list of molecular interactions can be seen in Supplementary Data S3.

Complex	CC	CO	CN	CX	OO	OX	NO	NN	NX	XX
BACE1_VER	1770	818	1028	336	91	117	254	145	102	0
BACE1_VERMOD-33	2454	1000	1083	132	63	39	190	108	33	0
BACE1_VERMOD-57	2332	903	1484	144	34	41	330	230	35	0
BACE1_VERMOD-9	1991	805	1216	224	57	75	271	178	60	0
BACE1_VERMOD-10	2170	1100	1216	97	139	39	320	162	29	0
BACE1_VERMOD-168	2431	1149	1428	258	107	81	332	195	63	0

**Table 4:** Frontier molecular orbital energies, HOMO–LUMO gap, and dipole moments of Verubecestat (VER) and its best-performing derivatives.

Molecule	HOMO (eV)	LUMO (eV)	Gap (eV)	Dipole (D)
VER	−6.20	−1.90	4.30	2.52
VERMOD-33	−5.85	−1.98	3.87	1.91
VERMOD-57	−6.45	−2.71	3.74	2.99
VERMOD-9	−6.08	−2.46	3.62	4.43
VERMOD-10	−5.79	−2.50	3.29	5.13
VERMOD-168	−5.67	−1.74	3.93	1.80

**Table 5:** MD simulation parameters of BACE1 complexes with Verubecestat (VER) and top-performing derivatives over 200 ns of simulations, including RMSD, RMSF, RoG, SASA, ligand–protein center-of-mass distance, and number of hydrogen bond interactions.

Complex	Average RMSD (nm)	Average RMSF (nm)	Average RoG (nm)	Average SASA (nm^2^)	Average Distance (nm)	Number of Hydrogen Bonds Between the Ligand-Receptor
BACE1 (Apo-protein)	0.15 ± 0.01	0.08 ± 0.04	2.11 ± 0.01	176.08 ± 3.40	N/A	N/A
BACE1_VER	0.21 ± 0.06	0.09 ± 0.06	2.10 ± 0.01	176.59 ± 3.44	1.11 ± 0.06	3.24 ± 0.78
BACE1_VERMOD-33	0.18 ± 0.02	0.11 ± 0.05	2.10 ± 0.01	176.52 ± 3.05	0.99 ± 0.03	4.27 ± 0.60
BACE1_VERMOD-57	0.37 ± 0.04	0.08 ± 0.06	2.11 ± 0.01	176.87 ± 2.94	1.38 ± 0.03	2.78 ± 1.30
BACE1_VERMOD-9	0.64 ± 0.07	0.07 ± 0.04	2.10 ± 0.01	176.37 ± 2.88	1.37 ± 0.07	2.74 ± 0.79
BACE1_VERMOD-10	0.26 ± 0.06	0.09 ± 0.04	2.11 ± 0.01	177.61 ± 2.79	1.18 ± 0.05	1.04 ± 0.92
BACE1_VERMOD-168	0.31 ± 0.14	0.09 ± 0.05	2.11 ± 0.01	179.22 ± 2.45	1.15 ± 0.05	3.05 ± 1.21

**Table 6:** MM/PBSA binding free energies (ΔG_binding) of Verubecestat (VER) and top-performing derivatives in complex with BACE1.

Complex	MM/PBSA Free Binding Energy ΔG_Binding (kcal/mol)
BACE1_VER	−35.33 ± 5.21
BACE1_VERMOD-33	−51.12 ± 4.99
BACE1_VERMOD-57	−43.85 ± 4.42
BACE1_VERMOD-9	−21.79 ± 5.31
BACE1_VERMOD-10	−33.77 ± 4.41
BACE1_VERMOD-168	−37.55 ± 6.41

**Table 7:** In silico pharmacokinetics and ADMET properties of Verubecestat (VER) and its top-performing derivatives.

Parameter	VER	VERMOD-33	VERMOD-57	VERMOD-9	VERMOD-10	VERMOD-168
Molecular Weight (g/mol)	409.42	410.83	414.40	469.54	440.48	499.57
Hydrogen Bond Acceptors (HBA)	8	6	9	9	10	10
Hydrogen Bond Donors (HBD)	2	2	2	2	2	2
cLogP	0.48	3.48	1.32	1.51	0.63	1.56
Total Surface Area	279.25	293.18	312.90	341.37	311.02	371.98
Polar Surface Area (PSA)	126.13	89.60	145.77	145.21	159.15	143.54
Relative PSA	0.34	0.25	0.34	0.31	0.38	0.30
Mutagenic	None	None	None	None	None	None
Tumorigenic	None	None	None	None	None	None
Reproductive Effective	None	None	None	None	None	None
Irritant	None	None	None	None	None	None
Shape Index	0.53	0.55	0.55	0.51	0.52	0.57
Molecular Flexibility	0.42	0.37	0.37	0.43	0.30	0.40
Molecular Complexity	0.85	0.87	0.89	0.92	0.94	0.92
Solvent Accessible Surface Area (SASA)	526.22	548.33	505.21	674.14	591.97	677.86
Hydrophobic Component of SASA (FOSA)	285.82	335.24	248.62	455.55	362.03	497.79
Hydrophilic Component of SASA (FISA)	176.87	127.11	230.17	191.89	229.93	156.92
Percent Human Oral Absorption	13.87	29.12	27.42	15.64	17.13	18.51
QPlogHERG	−5.36	−5.70	−7.13	−7.48	−6.69	−7.19
QPPCaco	3.23	9.58	1.01	2.33	1.01	4.99
QPlogBB	−0.23	0.27	−0.61	−0.80	−1.05	−0.55
QPPMDCK	3.03	13.03	5.41	3.35	3.39	4.92
QPlogKp	−8.23	−7.51	−9.02	−8.41	−9.02	−7.58
QPlogKhsa	−0.44	−0.17	−0.48	−0.41	−0.63	−0.50

## Data Availability

The original contributions presented in this study are included in the article/Supplementary Material. Further inquiries can be directed to the corresponding author(s).

## Supplementary Materials

The supporting information can be downloaded at: https://www.mdpi.com/article/10.3390/ijms262412143/s1.

## Abbreviations

The following abbreviations are used in this manuscript

AD	Alzheimer’s disease
ADMET	Absorption, distribution, metabolism, excretion, and toxicity
AIRs	Ambiguous interaction restraints
BACE1	β-site APP-cleaving enzyme 1
BBB	Blood–brain barrier
CNS	Central nervous system
DFT	Density functional theory
FEP	Free-energy perturbation
FMO	Frontier molecular orbital
GAFF2	General amber force field
HADDOCK	High ambiguity driven protein-protein docking
HBA	Hydrogen bond acceptor
HBD	Hydrogen bond donor
HOMO	Highest occupied molecular orbital
ICT	Intramolecular charge transfer
LBD	Ligand-binding domain
LUMO	Lowest unoccupied molecular orbital
MD	Molecular dynamics
MM/PBSA	Molecular mechanics/Poisson-Boltzmann surface area
NPT	Number of particles, pressure, and temperature
NVT	Number of particles, volume, and temperature
PME	Particle mesh Ewald
PRODIGY	Protein binding energy prediction
RMSD	Root mean square deviation
RMSF	Root mean square fluctuation
RoG	Radius of gyration
SASA	Solvent-accessible surface area
SCF	Self-consistent field
SPCE	Single point charge extended
SPR	Surface plasmon resonance
STP	Single-trajectory protocol
