# Supplementary material for: Rescuing Verubecestat: An Integrative Molecular Modeling and Simulation Approach for Designing Next-Generation BACE1 Inhibitors
Source: Int J Mol Sci. 2025 Dec 17;26(24):12143. doi: 10.3390/ijms262412143 (PMC12733122; doi:10.3390/ijms262412143)
Supplement: Supplementary file 1 [file ijms-26-12143-s001.zip › Supplementary Data S4 - Detailed RoG and SASA profiles for each simulated complex, including single-complex plots and stability analyses.pdf]

Supplementary Data S4

Detailed RoG and SASA profiles for each simulated complex, including single-complex plots and stability analyses.

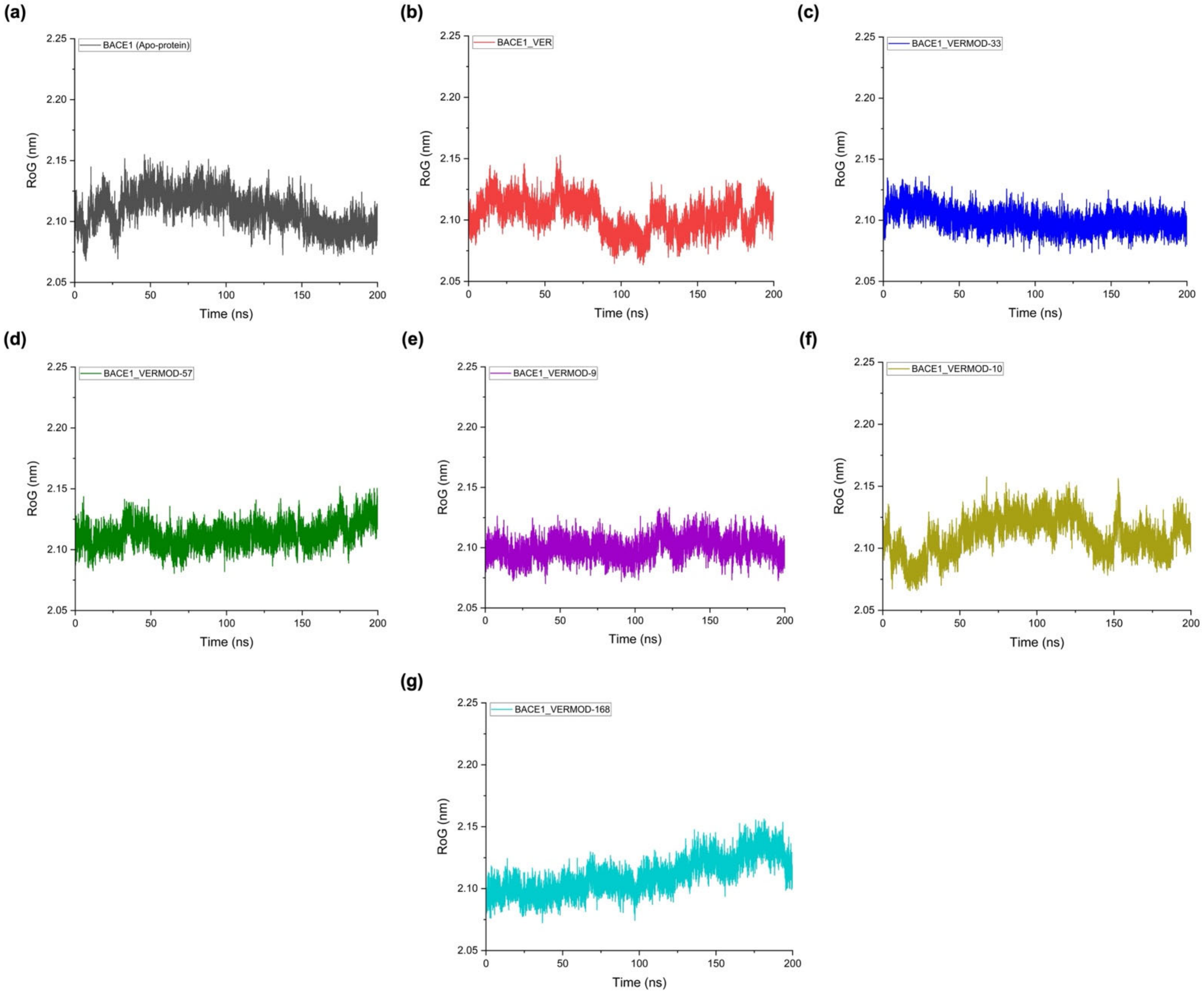

Supplementary Figure 1. Radius of gyration (RoG) plots. (**a**) BACE1 (Apo-protein). (**b**) BACE1_VER complex. (c) BACE1_VERMOD-33 complex. (**d**) BACE1_VERMOD-57 complex. (**e**) BACE1_VERMOD-9 complex. (**f**) BACE1_VERMOD-10 complex. (**g**) BACE1_VERMOD-168 complex.

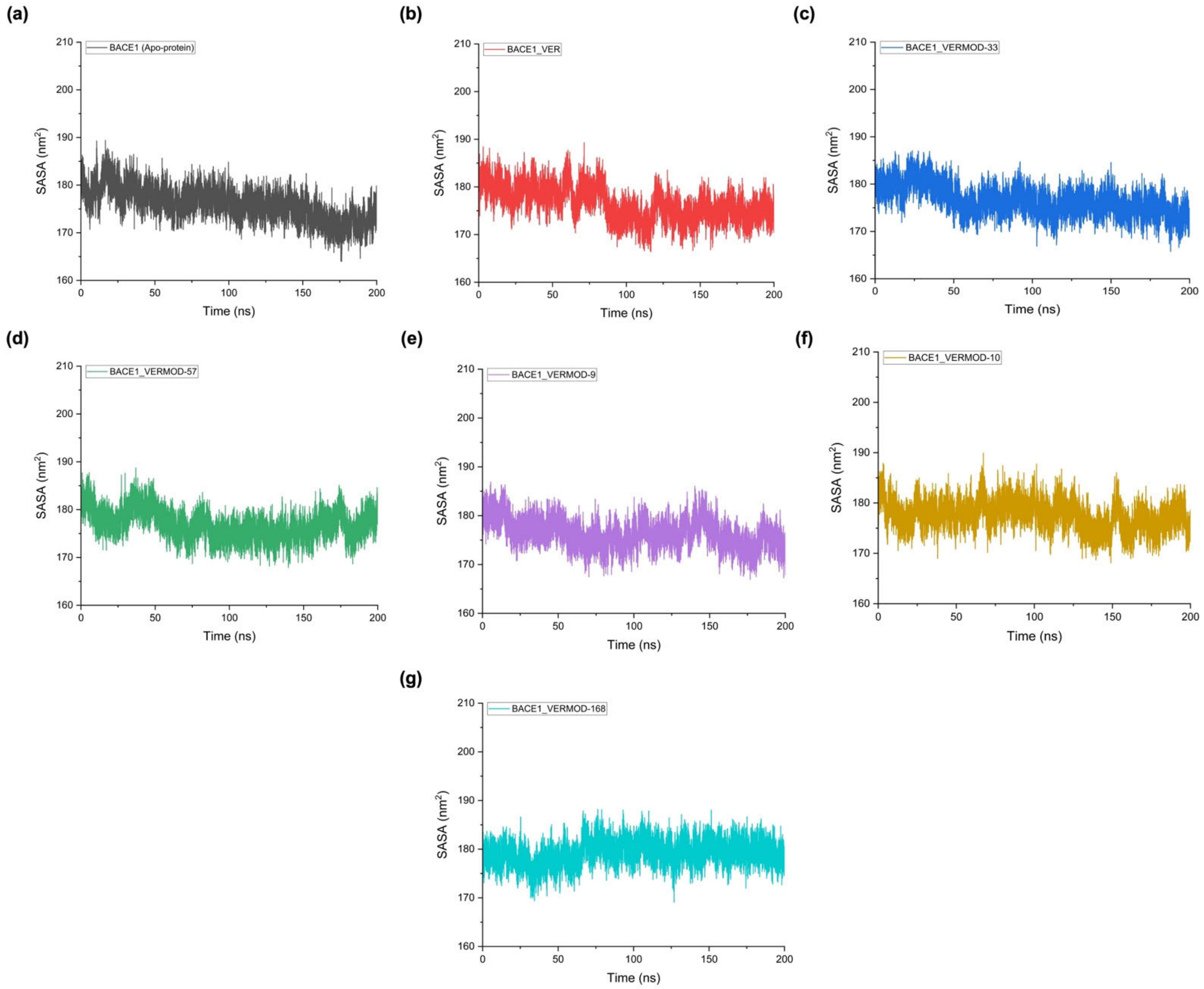

Supplementary Figure 2. Solvent-Accessible Surface Area (SASA) plots. (**a**) BACE1 (Apo-protein). (**b**) BACE1_VER complex. (c) BACE1_VERMOD-33 complex. (**d**) BACE1_VERMOD-57 complex. (**e**) BACE1_VERMOD-9 complex. (**f**) BACE1_VERMOD-10 complex. (**g**) BACE1_VERMOD-168 complex.
